# Changes in the Bacterial Community Structure of Remediated Anthracene-Contaminated Soils

**DOI:** 10.1371/journal.pone.0160991

**Published:** 2016-10-11

**Authors:** Laura Delgado-Balbuena, Juan M. Bello-López, Yendi E. Navarro-Noya, Analine Rodríguez-Valentín, Marco L. Luna-Guido, Luc Dendooven

**Affiliations:** Laboratory of Soil Ecology, ABACUS, Cinvestav, Mexico City, D.F., Mexico; Jinling Institute of Technology, CHINA

## Abstract

Mixing soil or adding earthworms (*Eisenia fetida* (Savigny, 1826)) accelerated the removal of anthracene, a polycyclic aromatic hydrocarbon, from a pasture and an arable soil, while a non-ionic surfactant (Surfynol^®^ 485) inhibited the removal of the contaminant compared to the untreated soil. It was unclear if the treatments affected the soil bacterial community and consequently the removal of anthracene. Therefore, the bacterial community structure was monitored by means of 454 pyrosequencing of the 16S rRNA gene in the pasture and arable soil mixed weekly, amended with Surfynol^®^ 485, *E*. *fetida* or organic material that served as food for the earthworms for 56 days. In both soils, the removal of anthracene was in the order: mixing soil weekly (100%) > earthworms applied (92%) > organic material applied (77%) > untreated soil (57%) > surfactant applied (34%) after 56 days. There was no clear link between removal of anthracene from soil and changes in the bacterial community structure. On the one hand, application of earthworms removed most of the contaminant from the arable soil and had a strong effect on the bacterial community structure, i.e. a decrease in the relative abundance of the Acidobacteria, Chloroflexi and Gemmatimonadetes, and an increase in that of the Proteobacteria compared to the unamended soil. Mixing the soil weekly removed all anthracene from the arable soil, but had little or no effect on the bacterial community structure. On the other hand, application of the surfactant inhibited the removal of anthracene from the arable soil compared to the untreated soil, but had a strong effect on the bacterial community structure, i.e. a decrease in the relative abundance of Cytophagia (Bacteroidetes), Chloroflexi, Gemmatimonadetes and Planctomycetes and an increase in that of the Flavobacteria (Bacteroidetes) and Proteobacteria. Additionally, the removal of anthracene was similar in the different treatments of both the arable and pasture soil, but the effect of application of carrot residue, earthworms or the surfactant on the bacterial community structure was more accentuated in the arable soil than in the pasture soil. It was found that removal of anthracene was not linked to changes in the bacterial community structure.

## Introduction

Contamination of soil with hydrocarbons is a recurring problem in petroleum producing countries such as Mexico. Some of these hydrocarbons, e.g. polycyclic aromatic hydrocarbons (PAHs), are toxic for humans and difficult to remove from soil [1]. Removal of these contaminants depends on their composition, availability and soil characteristics [2]. Removal of PAHs, a group of organic compounds with two to 13 aromatic rings, is slower from soil than that of alkanes and the more aromatic rings in the PAHs the longer the contaminant remains in soil [3]. Hydrocarbons are readily fixed on the soil matrix, i.e. organic material and minerals, which hampers their dissipation from soil [4].

Different techniques have been applied to remediate hydrocarbon-contaminated soil so as to limit the damage to the environment [5]. Some techniques try to increase the bioavailability of the contaminant while others try to stimulate microbial activity thereby accelerating the removal of the pollutant [6]. Surfactants, such as Surfynol^®^ 485, have been applied to soil to increase the bioavailability of PAHs in soil thereby accelerating their removal [7–9]. However, application of the surfactant can also inhibit the removal of PAHs from soil [7,8]. Organic material has often been applied to contaminated soil as it increases microbial activity and consequently the removal of the contaminant, but not always [10]. Applying earthworms to a contaminated soil increases microbial activity and the bioavailability of an organic contaminant [11]. The earthworm burrows through the soil increasing the contact between the contaminant and the soil microorganisms while the microbial activity in the gut of the earthworm is stimulated through the secretion of mucus and by the actively seeking of food by the earthworm [12]. Consequently, the removal of a contaminant is often accelerated in an earthworm-amended soil [13]. Regularly mixing the soil, which increased aeration and bioavailability of the contaminant, has shown to be a simply easy to apply technique to accelerate the removal of PAHs from soil [14,15].

Applying the above mentioned remediation techniques to an anthracene-contaminated soil, revealed that the removal rate was in the order: weekly mixing > applying earthworms > applying easily decomposable organic material > untreated soil > applying the surfactant such as Surfynol^®^ 485 [14]. Although abiotic factors affect the bioavailability of hydrocarbons in soil, mineralization of PAHs is mostly a biological process. Autochthonous soil microorganisms, mostly bacteria and fungi, are capable of mineralizing even complex PAHs, such as anthracene [16,17], but it remains unclear if the microbial community structure was also affected by the contaminant and the remediation techniques.

It has been shown that hydrocarbon contamination might have an effect on the bacterial community in soil [18], thereby reducing species richness, evenness, and phylogenetic diversity [19]. The question remains: are certain phylogenetic groups favoured or inhibited by the applied remediation techniques thus affecting the removal of the contaminant. To answer that question, two soils (an arable and a pasture soil) were spiked with anthracene and amended with organic material (carrot, *Daucus carota* ssp. *maximus* L.) that served as food for earthworms, the organic material plus the earthworm *Eisenia fetida* (Savigny, 1826), a non-ionic surfactant (Surfynol^®^ 485) or mixed regularly while the removal of the contaminant was monitored in an aerobic incubation experiment for 56 days. Anthracene, a PAH that consists of three fused benzene rings, has often been used as a model to study removal of PAHs from soil [20]. The bacterial community structure in soil was monitored by means of 454 pyrosequencing of the 16S rRNA gene. The objective of this research was to study how different strategies to remediate anthracene-contaminated soil affected the removal of the pollutant and the bacterial community structure in two different soils. The treatments applied to the soils, especially application of the surfactant and earthworms, had a strong effect on the bacterial community structure, but changes were not related to removal of anthracene, i.e. mixing soil weekly had little or no effect on the bacterial community structure but the removal of anthracene was the fastest in this treatment.

## Materials and Methods

### Chemicals used

Anthracene with purity >98% was obtained from Sigma-Aldrich (St. Louis, MO, USA) and acetone with purity >99.7% from J.T. Baker (Chesterfield, MO, USA). The non-ionic surfactant Surfynol^®^ 485 was obtained from Air Products and Chemicals de Mexico S.A. de C.V. (Mexico City, Mexico). It is an ethoxylated molecule of 2,4,7,9-tetramethyl-5-decyne-4,7-diol with 30 mol of ethylene oxide (EO) per molecule (C_14_H_14_(OH)_2_EO_30_) and a molecular weight of 1546 and a critical micelle concentration (CMC) of 11.2 mmol l^-1^ [21].

### Sampling site and soil sampling

Two soils were used in this study. An arable soil was collected in Otumba (State of Mexico, Mexico) (N.L. 19° 42’, W.L. 98° 49’), while a pasture soil was sampled in Juchique de Ferrer (State of Veracruz, Mexico) (N.L. 19° 50’, W.L. 96° 42’). Both fields that were sampled were privately owned and oral permission was given so that the soil could be collected. Details of the two sampling sites can be found in Delgado-Balbuena et al. [14].

Soil was sampled at random by augering 30 times the 0–15 cm top-layer of three plots of approximately 0.5 ha (S1 Fig). The soil from each plot was pooled and as such a total of six soil samples were obtained (three replicates of two soils). This field based replication was maintained in the laboratory study. The soil was taken to the laboratory on ice, 5-mm sieved and characterized.

### Experimental set-up

Details of how the two soils were contaminated with 500 mg anthracene using acetone can be found in Delgado-Balbuena et al. [14]. Five different treatments were applied to the anthracene-contaminated soil. In a first treatment, soil was amended with two adult *E*. *fetida* earthworms (0.35 g) obtained from INECOL (Xalapa, Veracruz, Mexico) and with a developed clitellum. The earthworms were fed 60 g carrot every two weeks. In a second treatment, soil was amended with 60 g organic material (carrot) every two weeks. As such, the effect of the earthworms on the removal of anthracene could be differentiated from that of the organic material applied. In a third treatment, soil was mixed for 10 min every 7 days. In a fourth treatment, soil was amended with 24.9 g kg^-1^ soil surfactant Surfynol^®^ 485 and mixed [9]. In a fifth treatment, soil was left unamended and served as control so that remediation capacity of the autochthonous microorganisms could be determined. Two more treatments were used in this study. In a first additional treatment, both soils were applied with the same amount of acetone used as carrier to contaminate the soil with anthracene and in a second additional treatment, unamended soil was used and served as control.

One hundred and five sub-samples of 500 g of both soils (soil replicates = 3, time of sampling = 5 (day 0, 3, 14, 28 and 56) and treatments applied = 7) treated as described above were added to polyvinyl chloride (PVC) tubes (diameter 10.5 cm, length 20 cm) containing a 5 cm layer of tezontle or porous volcanic rock. The amount of soil added to the PVC tubes was such that a 10 cm layer was obtained. The PVC columns were covered with perforated aluminium foil so that aeration was possible, but evaporation limited, and placed in a greenhouse. The soil water content was determined every other day as described in Delgado-Balbuena et al. [14]. After 3, 14, 28 and 56 days, a 20 g soil sample was taken from each column and extracted for anthracene [22] and DNA [23].

### Soil chemical analysis, DNA extraction and ribosomal libraries preparation

The soil samples were analyzed for particle size distribution by the hydrometer method, while total organic carbon was measured with a total organic carbon analyzer TOC-VCSN (Shimadzu, Canby, USA). Total nitrogen (N) was measured by the Kjeldahl method using concentrated H_2_SO_4_, K_2_SO_4_ and HgO to digest the sample. The water holding capacity (WHC) was determined by subtracting a given mass of a dry soil sample from the mass of the same sample saturated with water, left to drain overnight through Whatman No. 42 filter paper and covered with aluminum foil to avoid evaporation. The EC was measured in a saturated solution extract and pH in 1:2.5 soil-H_2_O suspension using a glass electrode (Table 1).

**Table 1:** Characteristics of the arable and pasture soil.

Salinity	pH	EC^a^ dS m^-1^	WHC^b^	Organic-C		Clay	Silt	Sand	USDA soil texture classification
			———————————(g kg^-1^ dry soil)——————————						
**Arable**	7.6	1.2	670		8.5	110	50	840	Sandy loam
	8.2	1.3	690		8.5	90	60	850	Sandy loam
	7.6	1.2	640		7.8	100	50	850	Sandy loam
**Pasture**	6.0	1.0	960		12.8	50	260	690	Loamy sand
	5.8	1.0	960		16.7	40	230	730	Loamy sand
	5.6	0.9	1030		17.7	50	220	730	Loamy sand

^a^ EC: Electrolytic conductivity

^b^ WHC: Water holding capacity.

The concentration of anthracene in the soil was determined using a modified exhaustive ultrasonic extraction method described by Song et al. [22]. Three g anhydrous sodium sulphate was mixed with a 1.5 g sub-sample of soil to form a fine powder. The mixture was added to a Pyrex tube, 10 mL acetone was applied and shaken mechanically on a vortex for 1 min. The tubes were placed in a sonicating bath at 30–40°C for 20 min. The extracts were centrifuged at 2000 × g for 7 min. The whole process of sonicating and centrifugation was repeated three times. The extracts were evaporated and dissolved in 1 mL acetone. Each sample was analyzed for anthracene on a Hewlett-Packard 4890–10 GC (Pennsylvania, USA) fitted with a flame ionization detector.

A HP-5 column from Hewlett-Packard with length 15 m, inner diameter 0.53 mm, and film thickness 1.5 μm was used to separate the anthracene with carrier gas He flowing at a rate of 7 ml/min. The oven temperature at 140°C was increased to 170°C at a rate of 2°C/min maintained at 170°C for 5 min. The temperature of the injector was 280°C and that of the detector 300°C. The detection limit of our GC analysis was 0.3 mg of anthracene per kg of dry soil. The amount of anthracene recovered with the exhaustive technique was 98%. Although the amount of anthracene lost during the procedure was <2%, data were adjusted for these small losses.

Each soil sample was washed first with 0.15 M sodium pyrophosphate and 0.15 M phosphate buffer pH 8 to remove the organic material before the DNA was extracted [24]. Three different methods were used to extract the DNA from soil. A first technique used a chemical and thermal shock for cell lysis [24]. In a second technique cells were enzymatically lysed [25] and in a third technique a detergent solution and mechanic disruption were used for cell lysis [26]. Each technique was used to extract four times 0.5 g soil per plot and pooled, i.e. a total of 6 g per plot. As such, a total of 18 g soil was extracted for DNA per soil (soil from three plots was used) and per treatment on each sampling day.

Primers 8–F (5´–AGA GTT TGA TCI TGG CTC A– 3´) and 556–R (5´ –TGC CAG IAG CIG CGG TAA– 3´), 10 pb multiplexed and containing the Roche 454 pyrosequencing adaptors Lib–L, were used to amplify the region V1–V3 of the 16S rRNA gene from the metagenomic DNA. Details of the PCR mixture and the thermal cycling scheme can be found in Navarro–Noya et al. [23]. All samples were amplified five times, pooled in equal amounts, and purified using the GFX^TM^ PCR DNA and Gel Band Purification Kit (GE Healthcare, UK) following the manufacturer instructions. Quantification of the PCR products was done using the NanoDrop^TM^ 2000 (Thermo Fisher Scientific Inc., Suwanee, GA, USA). Sequencing was done by Macrogen Inc. (DNA Sequencing Service, Seoul, Korea) by using a Roche GS–FLX Titanium 454 pyrosequencer (Roche, Mannheim, Germany) and the instructions of the manufacturer for amplicon sequencing.

### Analysis of pyrosequencing data

The QIIME version 1.8.0 software pipeline was used to analyse the pyrosequencing data [27]. Sequences were sorted by each multiplex-identifier and those < 200 bp in length, reads with < 25 quality threshold and containing any unresolved nucleotides were removed from the pyrosequencing–derived datasets. Chimeras were detected using ChimeraSlayer [28] and eliminated. Operational taxonomic units (OTU) were determined for the screened sequences by using the Uclust OTU picker at a similarity threshold of 97% and using an open reference strategy against the Greengenes core–set–aligned available at http://greengenes.lbl.gov/ [29]. The representative sequences for each of the clusters were aligned to the Greengenes core–set–aligned using PyNast [30]. The minimum percent sequence identity to include a sequence in the alignment was set at 75%. Taxonomy assignation was done by using the naïve Bayesian rRNA classifier from the Ribosomal Data Project (http://rdp.cme.msu.edu/classifier/classifier.jsp) [31] at a confidence threshold of 80%. The obtained biological observation matrix (BIOM) table was rarefied to 4,057 reads to avoid bias in diversity analysis by differences in sampling–sequencing effort. Diversity (Shannon, Simpson and Phylogenetic diversity indices) and species richness estimators (Chao1) were calculated using the rarified datasets within QIIME pipeline with the script alpha_rarefaction.py. The relative abundances were calculated for OTU and genus-taxonomic level in each sample.

### Phylogenetic and statistical analysis

The UniFrac analysis, to compare bacterial communities using phylogenetic information, was done within the QIIME pipeline [27], the resulting pairwise distance matrix of soil samples was clustered with a multivariate principal coordinate analysis (PCoA). An analysis of similarities (ANOSIM) was used to determine the effect of soil, organic material application and incubation time on the bacterial community based on weighted UniFrac pairwise distances. The effect of soil and treatment on the relative abundance of the bacterial groups at different taxonomic levels (phylum, class, order, family and genus) was determined with an analysis of variance (ANOVA) based on the least significant difference using the General Linear Model procedure [32]. Abundance of the bacterial groups (phylum, class, order, family and genus) was explored separately with a PCA using PROC FACTOR [32]. A canonical correlation analysis (CCA) was used to study the relationship between the abundance of the different bacterial groups and the soil characteristics of the arable and pasture soil. The CCA was done using the PROC CANCORR of the SAS statistical package [32].

## Results

### Sequences retrieved from soil, bacterial diversity and species richness

The number of sequences obtained from each soil sample varied between 4057 to 10545 in the arable soil (Table 2) and from 4494 to 13719 in the pasture soil (Table 3). The number of OTUs was > 400 in the arable and pasture soil except for the surfactant-amended arable soil when only 229 OTUs were extracted after 56 days. In the arable soil, the phylogenetic diversity and the Chao1 index were similar in each treatment, except for the surfactant-amended soil. In the surfactant-amended arable soil, the phylogenetic diversity and the Chao1 index were lower than in the other treatments and tended to decrease over time. In the pasture soil, the phylogenetic diversity and the Chao1 index were similar in each treatment.

**Table 2:** Alpha diversity parameters of the bacterial community in the arable soil.

Soil	Treatment	Day		Sequences	Phylogenetic diversity	Chao 1	Observed OTUs
**Arable**	Unamended		0	8729	41.9 (0.9)	1206 (41)	607 (17)
			3	9161	39.4 (4.0)	1129 (129)	546 (79)
			14	7445	38.4 (3.8)	1084 (152)	589 (85)
			28	7622	41.7 (1.1)	1307 (56)	630 (23)
			56	5220	38.4 (1.4)	1024 (74)	607 (19)
	Acetone		3	8214	41.0 (2.0)	1150 (77)	601 (31)
			14	8087	38.5 (0.6)	1102 (66)	586 (13)
			28	7266	43.0 (2.0)	1359 (56)	682 (15)
			56	6237	40.0 (2.3)	1142 (84)	620 (38)
	Anthracene		0	8383	43.1 (0.4)	1230 (22)	633 (7)
			3	7469	39.4 (1.9)	1196 (32)	563 (44)
			14	7150	39.9 (4.0)	1155 (154)	616 (72)
			28	7261	41.4 (1.1)	1289 (100)	643 (33)
			56	5933	38.0 (0.8)	1101 (21)	592 (3)
	Mixing		3	7905	40.4 (4.4)	1227 (198)	608 (83)
			14	6889	40.3 (0.7)	1156 (80)	617 (26)
			28	8995	42.0 (1.6)	1360 (101)	670 (41)
			56	8478	36.0 (1.4)	1030 (38)	534 (10)
	Organic material		3	8481	37.8 (3.0)	1032 (109)	517 (43)
			14	9765	35.7 (2.0)	962 (89)	511 (45)
			28	7861	41.1 (1.4)	1205 (77)	600 (34)
			56	7453	40.2 (2.0)	1088 (97)	576 (44)
	Earthworm		3	8675	36.5 (4.6)	1015 (79)	508 (83)
			14	10545	38.8 (2.1)	1158 (75)	570 (54)
			28	4057	37.5 (2.7)	1011 (191)	503 (60)
			56	7967	36.9 (2.6)	879 (88)	484 (31)
	Surfactant		3	8577	32.1 (2.0)	889 (53)	437 (29)
			14	9431	28.6 (3.9)	794 (125)	413 (72)
			28	7592	29.2 (4.4)	822 (136)	406 (61)
			56	10036	16.5 (0.3)	385 (33)	229 (19)

**Table 3:** Alpha diversity parameters of the bacterial community in the pasture soil.

Soil	Treatment	Day	Sequences	Phylogenetic diversity	Chao 1	Observed OTUs
**Pasture**	Unamended	0	8082	37.7 (0.0)	1086 (15)	566 (3)
		3	13719	39.9 (0.3)	1174 (47)	588 (5)
		14	9945	39.4 (1.4)	1185 (104)	622 (32)
		28	9976	41.4 (1.8)	1370 (66)	635 (39)
		56	7549	36.6 (2.1)	1000 (38)	544 (21)
	Acetone	3	10094	39.3 (0.9)	1145 (58)	584 (16)
		14	8979	38.1 (0.6)	1152 (45)	588 (29)
		28	10133	38.8 (0.9)	1298 (59)	600 (10)
		56	7856	37.2 (0.3)	1052 (45)	571 (19)
	Anthracene	0	8367	37.3 (1.4)	1036 (79)	558 (29)
		3	8539	38.5 (0.5)	1125 (69)	580 (6)
		14	10187	40.6 (1.4)	1178 (75)	611 (30)
		28	10492	40.3 (0.2)	1334 (12)	618 (4)
		56	4588	39.3 (0.9)	1142 (48)	524 (10)
	Mixing	3	6460	40.7 (0.1)	1187 (8)	615 (5)
		14	6631	37.8 (1.1)	1210 (79)	625 (22)
		28	11675	41.3 (0.7)	1413 (21)	637 (5)
		56	7683	34.2 (1.8)	958 (41)	527 (3)
	Organic material	3	7711	38.2 (0.9)	1069 (33)	561 (8)
		14	5768	38.2 (0.3)	1181 (30)	610 (2)
		28	8386	38.1 (1.3)	1137 (166)	579 (42)
		56	4494	33.9 (2.5)	885 (62)	493 (38)
	Earthworm	3	11728	39.4 (0.6)	1146 (22)	604 (12)
		14	6693	39.0 (0.1)	1058 (18)	596 (15)
		28	7410	40.0 (0.6)	1161 (179)	582 (28)
		56	5550	36.4 (1.4)	968 (75)	510 (22)
	Surfactant	3	7235	37.5 (0.9)	1051 (51)	563 (15)
		14	5677	36.8 (0.9)	1001 (7)	558 (16)
		28	8399	36.8 (1.5)	1082 (175)	556 (41)
		56	5636	34.2 (1.9)	924 (28)	497 (34)

### The bacterial community structure in the untreated pasture and arable soil

**Fig 1:**
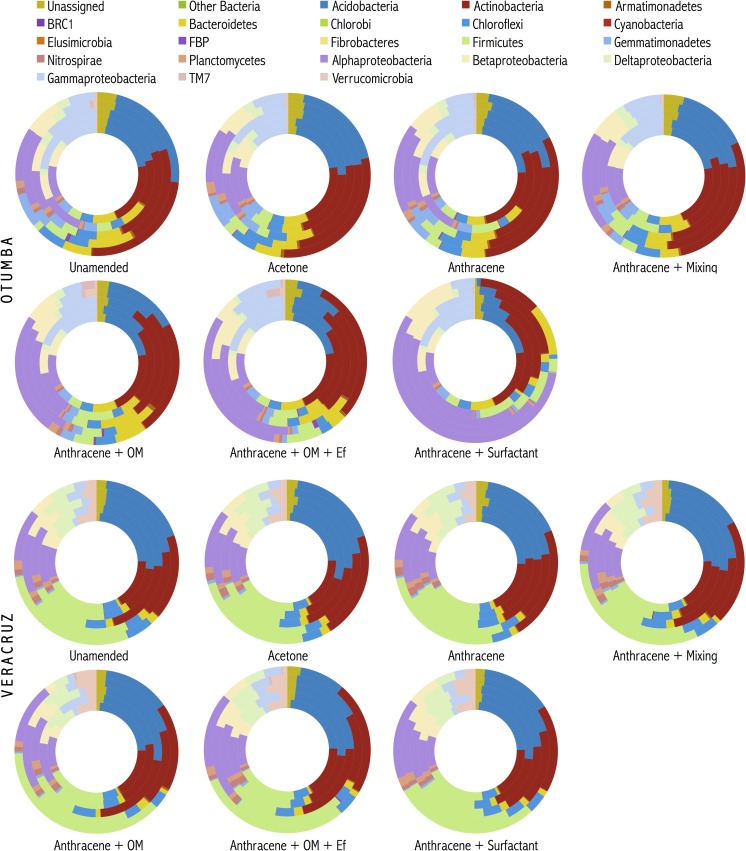
Changes in the relative abundance of the different bacterial phyla found in the arable Otumba soil and the pasture soil from Veracruz incubated aerobically for 56 days. Treatments are: unamended soil, acetone-amended, anthracene-amended, anthracene-amended and mixed every week, applied with anthracene plus organic material (OM), anthracene plus OM and the earthworm *Eisenia fetida* (Ef), and anthracene plus surfactant. Each ring within a figure represents a day of sampling, i.e. the inner ring is day 0, the second ring day 3, the third day 14, the fourth day 28 and the last day 56.

The relative abundance of the phyla that dominated in the arable and pasture soil was mostly similar (Fig 1). The Proteobacteria was the dominant phyla in both the arable (33.8±0.09%) and the pasture soil (25.8±0.04%), followed by the Actinobacteria (21.2±0.06%) in the arable soil and Acidobacteria (21.7±0.04%) in the pasture soil. The difference in bacterial population community between the two soils was due mostly to a higher relative abundance of the Gemmatimonadetes (4.4±0.02%) and Bacteroidetes (7.2±0.03%) in the arable soil compared to the pasture soil (0.7±0.004%) and (1.6±0.01%) and that of the Firmicutes (20.4±0.02%) and Verrucomicrobia (3.0±0.02%) in the pasture soil compared to the arable soil (3.9±0.004%) and (0.3±0.004%), respectively.

**Fig 2:**
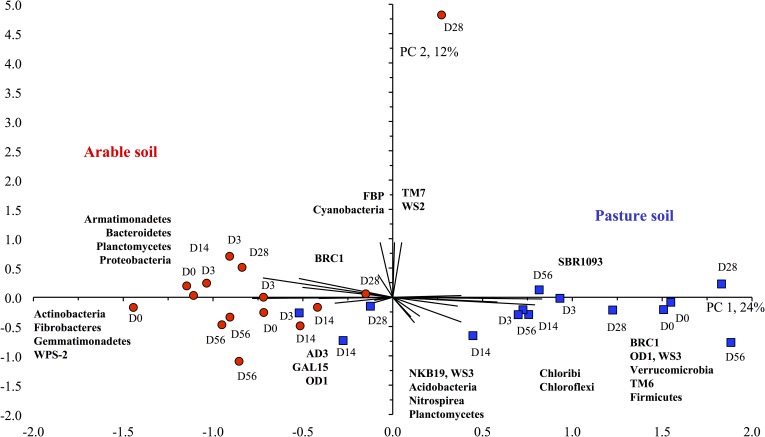
Principal component analysis. Principal component analysis with the relative abundance of the different bacterial phyla found in the pasture soil (◼, designated with a blue square) and arable soil (●, designated with a red circle) at the onset of the experiment (d0), incubated aerobically for 3 days (d3), 14 days (d14), 28 days (d28) or 56 days (d56).

**Fig 3:**
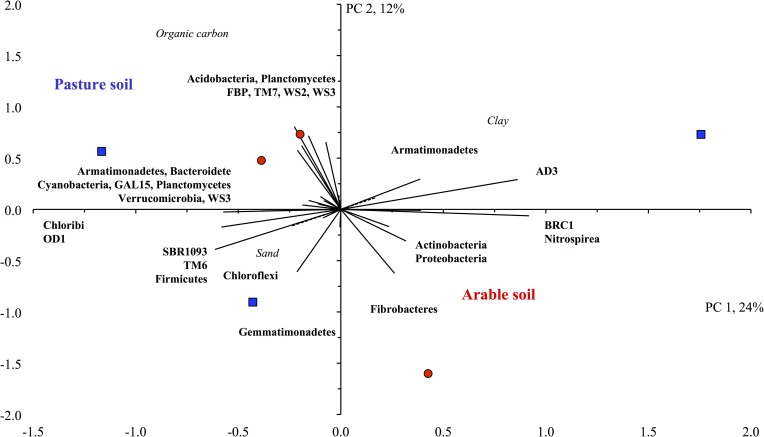
Canonical correlation analysis. Canonical correlation analysis with the relative abundance of the different bacterial phyla found in the pasture soil (◼,designated with a blue square) and arable soil (●, designated with a red circle) at the onset of the experiment (d0) and physic-chemical characteristics of the soils.

Consequently, the PCA considering the taxonomic distribution separated clearly the bacterial communities of the two soils (Fig 2) and the ANOSIM analysis considering the UniFrac distance matrices showed that the bacterial populations in both soils were highly significantly different (*P* < 0.001) (Table 4). A similar pattern emerged when a PCA was done with other taxonomic levels of the bacteria, i.e. class, order, family or genus (Data not shown). The two soils, however, were not separated clearly, considering the soil characteristics and the relative abundance of the bacterial groups (CCA) (Fig 3). This suggested that soil characteristics other than those measured in this study defined the bacterial populations in the two soils.

**Table 4:** ANOSIM analyses.

Comparison	*R*	*P*
**Arable versus Pasture soil**	0.7449	< 0.001
**Treatment**	0.0838	< 0.001
**Arable soil**		
**Treatment**	0.3408	< 0.001
**Incubation time**	0.1488	< 0.001
**Pasture soil**		
**Treatment**	0.0487	0.027
**Incubation time**	0.3052	< 0.001

Analyses based on weighted UniFrac pairwise distances, testing for differences in the bacterial communities between soils, treatment and incubation time.

### Removal of anthracene from the arable and pasture soil

**Fig 4:**
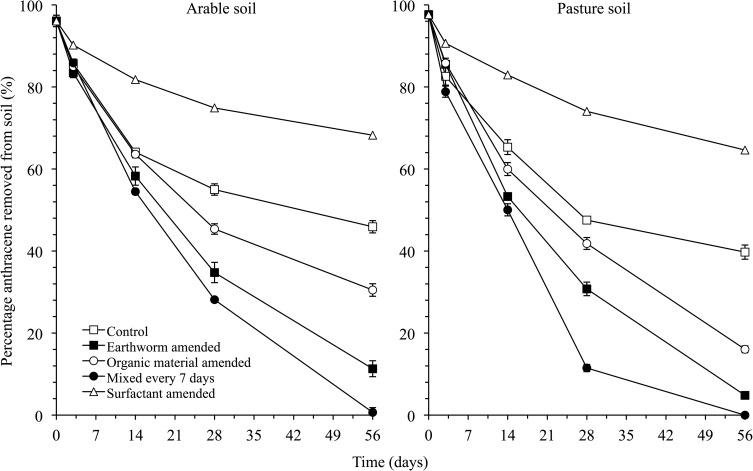
Percentage (%) removal of anthracene from soil. In the a) unamended pasture soil or b) in the unamended arable soil (◻), in soil amended with organic material (○), organic material and earthworms (◼) or surfactant (△), or mixed every seven days (●). Soil was incubated at 22±2°C for 112 days. Bars are ± one standard deviation.

The removal of the anthracene was different between the treatments, but similar in the arable and the pasture soil (Fig 4). Approximately 57% of the contaminant (mean of the two soils) was removed from the anthracene-amended soil within 56 days. Applying carrot residue increased the amount removed to 77% and earthworms plus the residue to 92%. Mixing the soil weekly removed all anthracene from both soils after 56 days. Applying surfactant, however, reduced the amount of anthracene removed to only 34%.

### The bacterial community structure in the arable soil

Application of anthracene had no significant effect on the relative abundance of the bacterial groups compared to the unamended soil, and the effect of application of acetone or mixing the soil on the bacterial community structure was minimal (Table 5). The relative abundance of the Xanthomonadales increased from 4.0±0.01% in the unamended soil to 6.0±0.02% in the acetone-amended soil and that of the Christensenellaceae (Firmicutes) from undetectable to 0.03±0.001%. The relative abundance of the Xanthomonadales increased from 4.0±0.01% in the unamended soil to 6.0±0.03% in the mixed soil and that of the Intrasporangiaceae (Actinobacteria) from 0.02±0.001% to 2.8±0.04% (*P* < 0.01). Details of these changes are given in the S2 Fig.

**Table 5:** Bacterial groups affected significantly by application of acetone or mixing the soil (i.e. compared to the untreated unamended control) in the arable soil incubated aerobically for 56 days.

Treatment	Phylum	Class	Order	Family	Genus
The relative abundance of the bacterial group was **higher** compared to the untreated control soil					
**Acetone**	Firmicutes	Clostridia	Clostridiales	Christensenellaceae*	
	Proteobacteria	Gammaproteobacteria	Xanthomonadales*		
**Mixed**	Actinobacteria	Actinobacteria	Actinomycetales	Intrasporangiaceae*	
	Proteobacteria	Gammaproteobacteria	Xanthomonadales*		

*Significant at the *P*<0.01

**Significant at the *P*<0.001

***Significant at the *P*<0.0001.

Application of surfactant to the arable soil had the largest impact on the bacterial community structure and reduced significantly the relative abundance of a wide range of bacterial groups, i.e. Acidobacteria, Cytophagia (Bacteroidetes), Chloroflexi, Gemmatimonadetes and Planctomycetes, compared to the unamended soil and significantly increased that of the Sphingobacteriia (Bacteroidetes) and Proteobacteria (*P* < 0.01) (Tables 6 and 7). These effects increased generally over time (Fig 1). The relative abundance of the Acidobacteria showed the largest reduction when surfactant was applied to soil, i.e. from 18.4±0.06% in the unamended soil to 4.3±0.03% in the surfactant-amended soil, while that of the Proteobacteria showed the largest increase, i.e. from 33.8±0.09% in the unamended soil to 61.3±0.13% in the surfactant-amended soil (*P* < 0.0001). The increase in the relative abundance of the latter was due mostly to an increase in the relative abundance of the genus *Sphingobium* (Alphaproteobacteria, Sphingomonadales), i.e. from 0.3±0.01% in the unamended soil to 17.2±0.14% in the surfactant-amended soil, while the decrease of the Acidobacteria was mostly due to the order iii1-15 (Acidobacteria-6), i.e. from 9.9±0.03% in the unamended soil to 1.8±0.02% in the surfactant-amended soil. Different groups of the Actinobacteria responded in different ways to the application of the surfactant. The relative abundance of the Rubrobacterales decreased from 5.5±0.03% and Gaiellales from 2.9±0.02% in the unamended soil to 3.0±0.01% and 1.5±0.01%, respectively, in the surfactant-amended soil, while that of the Actinomycetales increased from 9.3±0.05% in the unamended soil to 13.1±0.13% in the surfactant-amended soil.

**Table 6:** The relative abundance of the bacterial groups decreased significantly by application of surfactant compared to the untreated unamended control arable soil incubated aerobically for 56 days.

The relative abundance of the bacterial group was **lower** compared to the untreated control soil					
**Surfactan**t	Acidobacteria***	Acidobacteria-6***	iii1-15***	mb2424***	
		[Chloracidobacteria]***	RB41***		
		iii1-8***	DS-18***		
	Actinobacteria	Rubrobacteria***	Rubrobacterales***	Rubrobacteraceae***	Rubrobacter***
		Thermoleophilia***	Gaiellales**	Gaiellaceae**	
	Bacteroidetes	Cytophagia***	Cytophagales***	Cytophagaceae***	Adhearibacter***
		[Saprospirae]	[Saprospirales]***	Chitinophagaceae***	Flavisolibacter***
					Segetibacter**
	Chloroflexi***	Anaerolineae***			
		Chloroflexi***	[Roseiflexales]***	[Kouleothrixaceae]***	Kouleothrix***
		S085*			
		TK10*			
	Gemmatimonadetes***	Gemm-3**			
		Gemm-5**			
		Gemmatimonadetes***	Ellin5290***		
			Gemmatimonadales***	Ellin5301***	
			N1423WL***		
	Nitrospirae	Nitrospira	Nitrospirales	Nitrospiraceae*	
	Planctomycetes**	Phycisphaerae***	WD2101***		
	Proteobacteria	Betaproteobacteria	Ellin6067*		
			MND1**		
		Deltaproteobacteria***	Myxococcales***		

*Significant at the *P*<0.01

**Significant at the *P*<0.001

***Significant at the *P*<0.0001.

**Table 7:** The relative abundance of the bacterial groups increased significantly by application of surfactant compared to the untreated unamended control arable soil incubated aerobically for 56 days.

**Surfactant**	Actinobacteria	Actinobacteria***	Actinomycetales***	Mycobacteriaceae ***	Mycobacterium***
				Streptomycetaceae*	
	Bacteroidetes*	Sphingobacteriia**	Sphingobacteriales**	Sphingobacteriaceae***	Olivibacter**
	Proteobacteria***	Alphaproteobacteria***	Rhizobiales***	Bradyrhizobiaceae***	Balneimonas***
					Bosea***
				Rhizobiaceae***	Agrobacterium***
			Caulobacterales	Caulobacteraceae	Phenylobacterium*
			Sphingomonadales***	Sphingomonadaceae***	Sphingobium***
		Betaproteobacteria	A21b*		
			Burkholderiales*	Burkholderiaceae	Burkholderia*
				Oxalobacteraceae***	Cupriavidus***
		Gammaproteobacteria	Xanthomonadales	Xanthomonadaceae*	Pseudoxanthomonas*

*Significant at the *P*<0.01

**Significant at the *P*<0.001

***Significant at the *P*<0.0001.

Addition of earthworms had also a large impact on the bacterial community structure in the arable soil, but less bacterial groups were affected and the effect was less strong than when surfactant was applied to soil. Additionally, the effect was less pronounced over time (Fig 1). Earthworms reduced significantly the relative abundance of the Acidobacteria, Chloroflexi and Gemmatimonadetes compared to the unamended soil and significantly increased that of the Proteobacteria (Table 8). For instance the relative abundance of the Acidobacteria decreased from 18.4±0.06% in the unamended soil to 9.2±0.05% in the earthworm-amended soil, while that of the Proteobacteria increased from 33.8±0.09% in the unamended soil to 43.8±0.06% in the earthworm-amended soil. The relative abundance of the same acidobacterial group was reduced (iii1-15) in the earthworm-amended soil as when surfactant was applied to soil. Within the Alphaproteobacteria, the largest increase was found for the Rhodobacterales. Their relative abundance increased from 0.5±0.001% in the unamended soil to 4.5±0.05% in the earthworm-amended soil.

**Table 8:** Bacterial groups affected significantly by application of earthworms (i.e. compared to the untreated unamended control) in the arable soil incubated aerobically for 56 days.

The relative abundance of the bacterial group was **lower** compared to the untreated control soil					
Acidobacteria***	Acidobacteria-6***	iii1-15***		
	iii1-8***	DS-18***		
Actinobacteria	Rubrobacteria***	Rubrobacterales***	Rubrobacteraceae***	Rubrobacter***
	Thermoleophilia***	Gaiellales**	Gaiellaceae**	
Bacteroidetes	[Saprospirae]	[Saprospirales]***	Chitinophagaceae***	Flavisolibacter***
Chloroflexi***	Chloroflexi***	[Roseiflexales]***	[Kouleothrixaceae]***	
	S085*			
Gemmatimonadetes***	Gemm-5**			
	Gemmatimonadetes***	Ellin5290***		
		Gemmatimonadales***		
The relative abundance of the bacterial group was **higher** compared to the untreated control soil					
Actinobacteria	Actinobacteria***	Actinomycetales***	Microbacteriaceae***	Agromyces***
				Microbacterium***
			Micrococcaceae***	
			Nocardiaceae***	Rhodococcus***
			Promicromonosporaceae***	Cellulosimicrobium***
Bacteroidetes	Flavobacteria**	Flavobacteriales**	Flavobacteriaceae***	
Firmicutes	Bacilli	Bacillales	Paenibacillaceae*	
		Lactobacillales***	Leuconostocaceae***	Leuconostoc***
	Clostridia	Clostridiales	Lachnospiraceae**	Coprococcus**
			Veillonellaceae	Pelosinus*
Proteobacteria***	Alphaproteobacteria***	Rhizobiales***	Rhizobiaceae***	Kaistia***
		Rhodobacterales***	Rhodobacteraceae***	Amaricoccus***
				Rhodobacter*
		Rhodospirillales	Acetobacteraceae***	Gluconobacter***
	Betaproteobacteria	Nitrosomonadales*		
	Gammaproteobacteria	Alteromonadales	Alteromonadaceae***	Cellvibrio***
		Pseudomonadales	Pseudomonadaceae***	Pseudomonas***
		Xanthomonadales	Xanthomonadaceae	Stenotrophomonas*

*Significant at the *P*<0.01

**Significant at the *P*<0.001

***Significant at the *P*<0.0001.

Application of organic material affected similar bacterial groups as the application of earthworms, but less bacterial groups and to a lesser extent (Table 9, Fig 1). For instance, the relative abundance of the Rubrobacterales decreased from 5.5±0.03% in the unamended soil to 2.2±0.01% in the earthworm-amended soil and 2.9±0.01% in the organic material amended soil, while that of the Actinomycetales increased from 9.3±0.05% to 19.6±0.03% in the earthworm-amended soil and to 17.1±0.06% in the organic material amended soil.

**Table 9:** Bacterial groups affected significantly by application of organic material (i.e. compared to the untreated unamended control) in the arable soil incubated aerobically for 56 days.

The relative abundance of the bacterial group was **lower** compared to the untreated control soil				
Acidobacteria***	Acidobacteria-6***	iii1-15***		
	iii1-8***	DS-18***		
Actinobacteria	Rubrobacteria	Rubrobacterales***	Rubrobacteraceae***	Rubrobacter***
Chloroflexi***				
Gemmatimonadetes***	Gemmatimonadetes***	Ellin5290***		
Proteobacteria	Betaproteobacteria	MND1**		
The relative abundance of the bacterial group was **higher** compared to the untreated control soil				
Actinobacteria	Actinobacteria***	Actinomycetales***	Micrococcaceae***	
			Nocardiaceae***	Rhodococcuss***
			Nocardioidaceae	Nocardioides**
				Pimelobacter*
Proteobacteria	Alphaproteobacteria	Rhodospirillales*	Acetobacteraceae***	Gluconobacter***
				Roseococcus*
		Caulobacterales	Caulobacteraceae	Mycoplana*
	Gammaproteobacteria	Pseudomonadales	Pseudomonadaceae***	Azorhizophilus*
				Pseudomonas***

*Significant at the *P*<0.01

**Significant at the *P*<0.001

***Significant at the *P*<0.0001.

**Fig 5:**
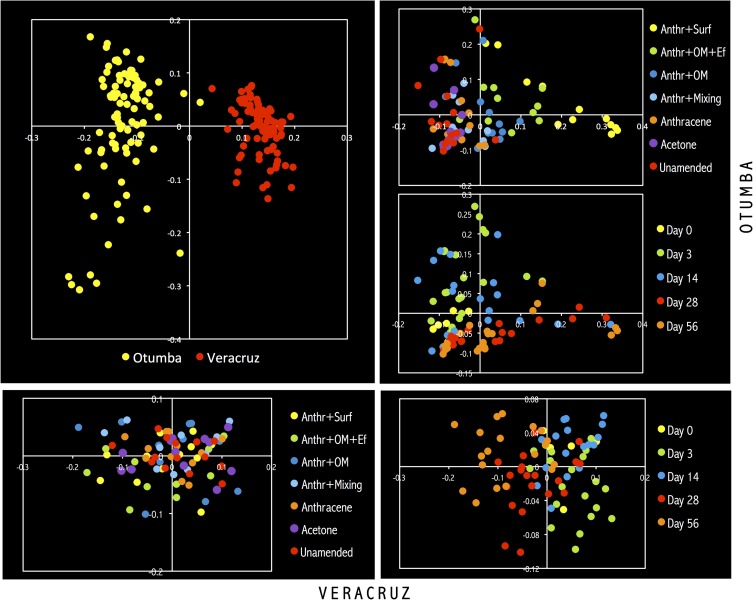
Weighted Unifrac analysis of the different OTUs found in the arable Otumba soil and the pasture soil from Veracruz incubated aerobically for 56 days. Treatments are: unamended soil, soil applied with acetone (Acetone), anthracene (Anthracene), anthracene and mixed every week (Anthr+Mixing), applied with anthracene plus organic material (Anthr+OM), anthracene plus organic material plus the earthworm *Eisenia fetida* (Anthr+OM+Ef) and anthracene plus surfactant (Anthr+Surf). Soil was incubated aerobically for 0 days (Day 0), 3 days (Day 3), 14 days (Day 14), 28 days (Day 28) or 56 days (Day 56).

**Fig 6:**
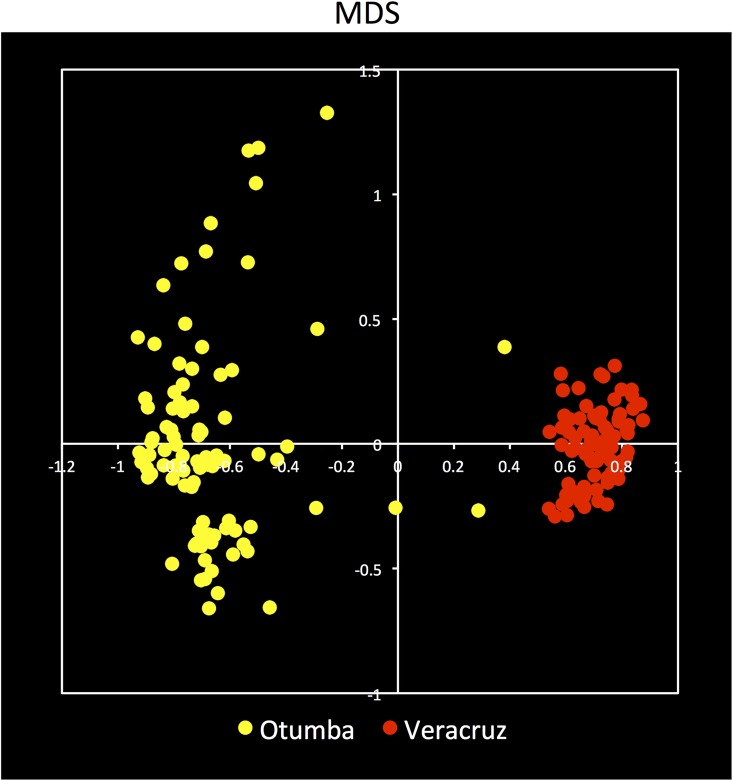
Principal component analysis with the relative abundance of the different bacterial orders found in the arable soil. Treatments are: unamended soil (◼, designated with a red square), soil applied with acetone (◼, designated with a violet coloured square), anthracene (◼, designated with a brown square), anthracene and mixed every week (◼, designated with a yellow square), applied with anthracene plus organic material (◼, designated with a green square), anthracene plus organic material plus the earthworm *Eisenia fetida* (◼, designated with a light blue square) and anthracene plus surfactant (Anthr+Surf) (◼, designated with a blue square). Soil was incubated aerobically for 0 days (d0), incubated aerobically for 3 days (d3), 14 days (d14), 28 days (d28) or 56 days (d56).

**Fig 7:**
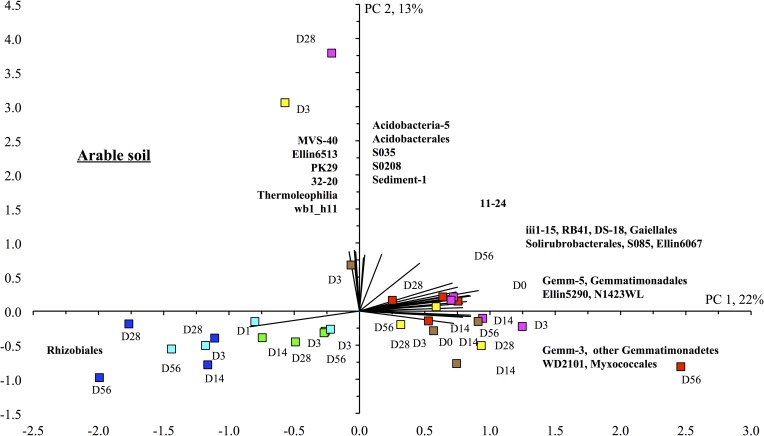
nMDS analysis of the different OTUs found in the arable Otumba soil and the pasture soil from Veracruz incubated aerobically for 56 days. Treatments are: unamended soil, soil applied with acetone (Acetone), anthracene (Anthracene), anthracene and mixed every week (Anthr+Mixing), applied with anthracene plus organic material (Anthr+OM), anthracene plus organic material plus the earthworm *Eisenia fetida* (Anthr+OM+Ef) and anthracene plus surfactant (Anthr+Surf). Soil was incubated aerobically for 0 days (Day 0), 3 days (Day 3), 14 days (Day 14), 28 days (Day 28) or 56 days (Day 56).

The PCA, weighted UniFrac and nMDS analysis confirmed the earlier mentioned effects of the different treatments on the bacterial community structure independent of the bacterial level considered (Figs 5, 6 and 7). Application of acetone, anthracene or mixing the soil had little or no effect on the bacterial community structure compared to the unamended soil (Fig 6). For instance, the relative abundance of Gemm-3, Gemm-5, Gemmatimonadales (Gemmatimonadetes), the iii1-15, RB41 and DS-18 (Acidobacteria), Gaiellales and Solirubrobacterales (Actinobacteria) and Myxococcales (Deltaproteobacteria) was larger in these treatments than in the other treatments. The carrot residue amended soil was separated from the unamended soil as the relative abundance of the Rhizobiales increased, and the effect was larger with addition of earthworms. Application of surfactant had the largest effect on the bacterial community structure compared to the unamended soil. The ANOSIM analysis showed also that the bacterial populations were affected significantly by treatment and incubation time (*P* < 0.001) (Table 4).

### The bacterial community structure in the treated pasture soil

In contrast to the arable soil, the relative abundance of less bacterial groups was affected by treatment and the effect was less profound in the pasture soil (Table 10; Fig 1). Additionally, the effect of application of earthworms on the bacterial community structure was more outspoken than that of surfactant. Only the relative abundance of the Alphaproteobacteria, TM7-3 and Verrucomicrobiae increased significantly when earthworms were applied to the pasture soil and Alphaproteobacteria when surfactant was applied (*P* < 0.05).

**Table 10:** Bacterial groups affected significantly by treatment (i.e. compared to the untreated unamended control) in the arable incubated aerobically for 56 days.

Treatment	Phylum	Class	Order	Family	Genus
The relative abundance of the bacterial group was **lower** compared to the untreated control pasture soil					
**Earthworm**	Chloroflexi †				
The relative abundance of the bacterial group was **higher** compared to the untreated control pasture soil					
**Mixed**	Actinobacteria	Actinobacteria	Actinomycetales	Streptosporangiaceae †	
				Intrasporangiaceae	Janibacter**
	Chloroflexi	Anaerolineae	SJA-15 †		
	Nitrospirae	Nitrospira	Nitrospirales	FW	4–29 †
**Organic material**	Firmicutes	Bacilli	Lactobacillales***	Leuconostocaceae***	Leuconostoc***
	Proteobacteria	Alphaproteobacteria	Rhodospirillales	Acetobacteraceae	Gluconobacter*
**Earthworm**	Actinobacteria	Actinobacteria	Actinomycetales	Microbacteriaceae*	Microbacterium**
				Micrococcaceae*	
	Firmicutes	Bacilli	Bacillales	Paenibacillaceae*	Paenibacillus***
			Lactobacillales***	Leuconostocaceae***	Leuconostoc***
		Clostridia	Clostridiales	Lachnospiraceae***	Coprococcus***
	Proteobacteria	Alphaproteobacteria †	Rhizobiales	Hyphomicrobiaceae	Devosia**
				Rhizobiaceae	
			Rhodospirillales	Acetobacteraceae***	
		Betaproteobacteria	Burkholderiales	Alcaligenaceae	Achromobacter*
				Comamonadaceae	Comamonas*
	TM7	TM7-3*			
	Verrucomicrobia	Verrucomicrobiae***	Verrucomicrobiales***	Verrucomicrobiaceae***	Luteolibacter***
**Surfactant**	Proteobacteria	Alphaproteobacteria †	Rhizobiales	Bradyrhizobiaceae †	
		Betaproteobacteria	Burkholderiales	Oxalobacteraceae	Cupriavidus**
		Gammaproteobacteria	Pseudomonadales	Pseudomonadaceae	Pseudomonas***

†Significant at the *P*<0.05

*Significant at the *P*<0.01

**Significant at the *P*<0.001

***Significant at the *P*<0.0001

**Fig 8:**
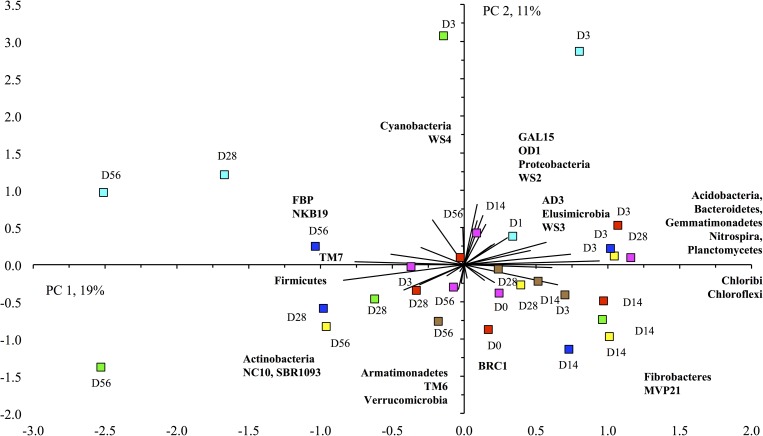
Principal component analysis with the relative abundance of the different bacterial phyla found in the pasture soil. Figure captions can be found in Fig 6. Soil was incubated aerobically for 0 days (d0), 3 days (d3), 14 days (d14), 28 days (d28) or 56 days (d56).

As mentioned before, the changes in the relative abundance of the different groups was much smaller in the pasture than in the arable soil. For instance, relative abundance of the Alphaproteobacteria increased from 11.0±0.02% in the unamended soil to 14.3±0.03% in the earthworm-amended soil and to 12.4±0.03% in the surfactant amended soil. Consequently the PCA and weighted UniFrac did not separate the different treatments clearly (Figs 5, 7 and 8). The ANOSIM analysis showed, however, that the bacterial populations were affected significantly by treatment although to a lower degree than in the arable soil (*P* < 0.027) (Table 4). However, incubation time, especially in the earthworm-amended soil, appears to affect the bacterial community structure. For instance, the relative abundance of the Firmicutes and Actinobacteria increased with incubation time (day 28 and 56) in the earthworm, carrot residue and surfactant amended soil. Consequently, the ANOSIM analysis showed that the bacterial populations were highly significantly different over time (*P* < 0.001) (Table 4).

### Changes in the bacterial groups favoured by the different treatments over time

Degradation of anthracene was most accentuated in the first days after its application to soil, but the removal of the contaminant in soil continued until day 56 and this affected the relative abundance of different bacterial groups. Considering the ratio between the relative abundance of the bacteria in the untreated soil and the treated soil, i.e. [(relative abundance of the bacterial group in the treated soil)- (relative abundance of the bacterial group in the untreated soil)]/ (relative abundance of the bacterial group in the untreated soil)*100, it can be derived how the different bacterial groups where affected by treatment. A positive ratio means an increase in relative abundance of the bacterial group as a result of the treatment applied to soil compared to the untreated soil or a negative value means a decrease in relative abundance.

**Fig 9:**
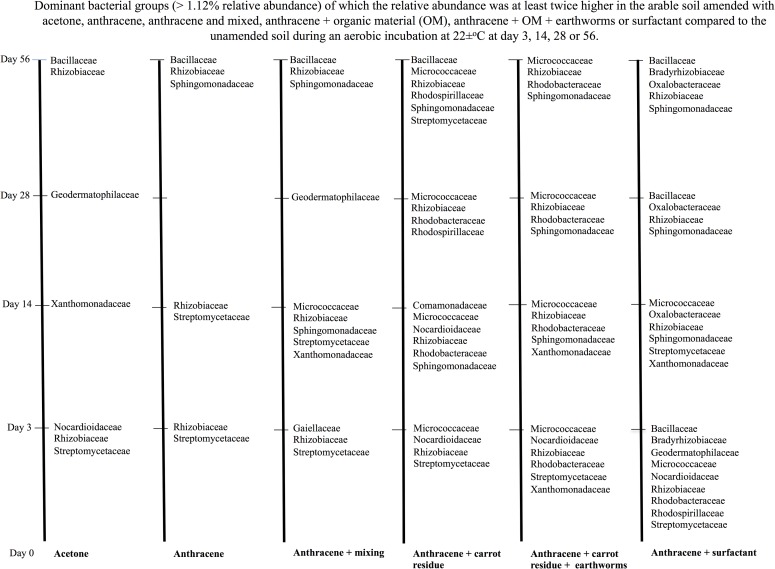
Bacterial groups affected positively by treatment in the arable soil. Treatments were application of acetone, acetone plus anthracene, acetone plus anthracene mixed weekly, acetone plus anthracene and organic material, acetone plus anthracene and organic material and earthworms, or acetone plus anthracene and surfactant compared to the unamended soil during an aerobic incubation of 56 days.

Considering only the most important bacterial groups, i.e. relative abundance ≥1.0%, that were favoured, i.e. their relative abundance ≥ doubled when soil was treated compared to the untreated soil, then Nocardiaceae and Streptomycetaceae (mostly *Streptomyces*, Actinomycetales), Rhizobiaceae (Alphaproteobacteria) were the first, i.e. at day 3, important degraders of the acetone and/or its metabolic products in the arable soil (Fig 9, and S3, S4, S5 and S6 Figs). They were replaced by Xanthomonadaceae at day 14, Geodermatophilaceae (Actinomycetales) at day 28 and finally by Bacillaceae (mostly *Bacillus*, Firmicutes) and Rhizobiaceae at day 56.

Application of anthracene with acetone as solvent favoured similar bacterial groups as in the acetone amended soil at day 3 (Fig 9 and S3, S4, S5 and S6 Figs). At day 14, degraders of acetone plus anthracene and/or its metabolic products were *Streptomyces* and Rhizobiaceae. *Streptomyces* was replaced by *Bacillus* and Sphingomonadaceae (mostly *Sphingobium*, Alphaproteobacteria) at day 56, although Rhizobiaceae remained an important degrader of anthracene and/or its metabolic products.

More bacterial groups were favoured when the acetone plus anthracene amended soil was mixed daily and that was most noticeable at day 14 (Fig 9 and S3, S4, S5 and S6 Figs). The first degraders of acetone plus anthracene remained *Streptomyces* and Rhizobiaceae, but phylotypes belonging to Gaiellaceae at day 3 and Micrococcaceae, Sphingomonadaceae and Xanthomonadaceae at day 14 also participated in the removal of anthracene and/or its metabolic products. They were replaced by Geodermatophilaceae as the most important degraders at day 28 and the latter by *Bacillus*, Rhizobiaceae and Sphingomonadaceae at day 56.

Application of organic material or/and earthworms further increased the bacterial groups that were favoured (Fig 9 and S3, S4, S5 and S6 Figs). Apart from *Streptomyces* and Rhizobiaceae, first degraders of the anthracene plus organic material were phylotypes that belonged to the Micrococcaceae and Nocardioidaceae and additionally Rhodobacteraceae and Xanthomonadaceae when earthworms were also added. Rhodospirillaceae were favoured towards the end of the incubation in the organic material amended soil, but not or far less when earthworms were also added.

Intriguingly, application of surfactant favoured even more bacterial groups although the removal of anthracene was lower than in the other treatments (Fig 9 and S3, S4, S5 and S6 Figs). Phylotypes belonging to the Oxalobacteraceae and Bradyrhizobiaceae were favoured specifically by the application of the surfactant.

**Fig 10:**
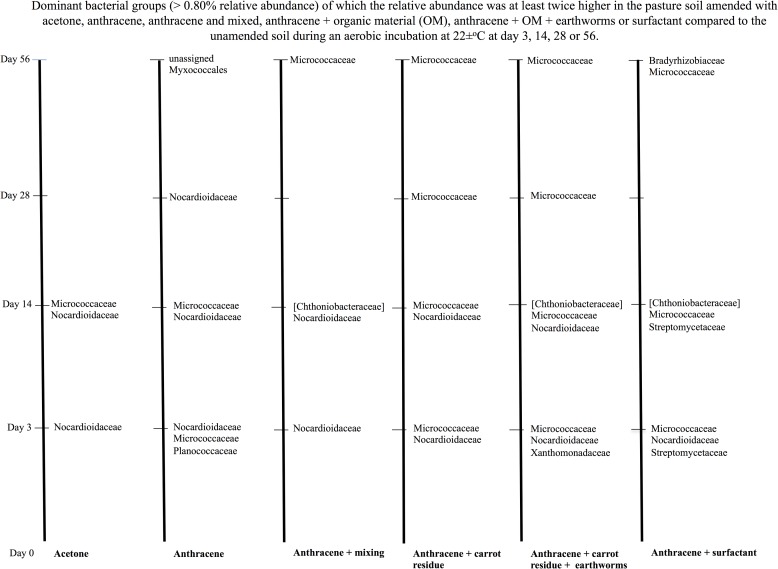
Bacterial groups affected positively by treatment in the pasture soil. Treatments were application of acetone, acetone plus anthracene, acetone plus anthracene mixed weekly, acetone plus anthracene and organic material, acetone plus anthracene and organic material and earthworms, or acetone plus anthracene and surfactant compared to the unamended soil during an aerobic incubation of 56 days.

The number of different bacterial groups of which the relative abundance was favoured by the different treatments was smaller in the pasture than in the arable soil and the effect was also generally smaller (Fig 10 and S7, S8, S9 and S10 Figs). Overall, similar bacterial groups were favoured by the different treatments in the pasture soil as in the arable soil, i.e. generally Nocardioidaceae and Micrococcaceae, except for the [Chthoniobacteraceae] and the Planococcaceae.

## Discussion

### Removal of anthracene from soil

The removal of anthracene was similar in the pasture and arable soil. After 56 days, 56% of the anthracene was removed from the unamended soil. Autochthonous microorganisms are well known to remove PAHs from soil [33]. All treatments applied to soil increased the removal of the contaminant, except for the application of surfactant. Applying carrot residue to the arable and the pasture soil increased the removal of anthracene to 77% after 56 days. It is well known that organic material stimulates microbial activity and enzymes will be released that might participate in the degradation of the contaminant [34]. Consequently the removal of anthracene from soil will be accelerated. Application of earthworms to the soil further increased the removal of anthracene from soil to 92% after 56 days. The earthworms burrow through the soil to feed on organic material. The soil is mixed in the gut of the earthworms thereby increasing the contact between the contaminant and the microorganisms, and accelerating the dissipation of the anthracene. Earthworms are also known to alter the microbial population in soil, which might increase the removal of the contaminant from soil [35]. Mixing the soil weekly, removed all anthracene from soil after 56 days. Mixing the soil increased the contact between the contaminant and the soil microorganisms and thus the removal of the contaminant from soil [14]. Application of surfactant, however, decreased the removal of anthracene from both soils in this study. In a previous experiment, application of the same surfactant increased the removal of anthracene and the microbial activity in a loamy sand soil [9]. It is difficult to predict what the effect of the application of a surfactant on the removal of a contaminant will be. The surfactant might affect the bioavailability of the contaminant and/or the microbial activity, thereby stimulating or inhibiting the dissipation of the pollutant.

### Bacterial community structure as affected by treatment

In both soils, application of acetone, acetone plus anthracene, or mixing the soil had only a limited or no effect on the bacterial community structure. Brinch et al. [36] suggested to mix the solvent (acetone) with 25% of the soil sample, followed by evaporation of the solvent and then to mix this 25% of the soil sample with the rest of the soil sample to minimize the effect of acetone on the indigenous soil microorganisms. This procedure was followed in this experiment and the effect of acetone on the bacterial community was small, i.e. only the relative abundance of the Xanthomonadales was higher in the acetone-amended arable soil than in the unamended arable soil. Contaminating the soil with anthracene had no significant effect on the bacterial community structure in both soils. In a previous experiment, contaminating an alkaline saline soil with electrolytic conductivity (EC) of 56 dS m^-1^ soil did alter the bacterial community structure so it can be speculated that soil characteristics might determine a possible effect of the pollutant [37]. The removal of anthracene increased after mixing, but the effect on the bacterial community structure was small. Only the relative abundance of a limited group of bacteria was higher in the mixed arable and pasture soils than in the untreated soils, e.g. Xanthomonadales in the arable soil and Streptosprangiaceae and *Janibacter* (Actinobacteria) in the pasture soil. O'Neill et al. [38] found that disturbance of a polar soil had also little or no effect on the bacterial community structure as found after mixing the soil in this experiment.

In the arable soil, application of surfactant, carrot residue and earthworms altered the bacterial community structure, but the effect was much smaller in the pasture soil. The relative abundance of the Acidobacteria, Chloroflexi and Gemmatimonadetes decreased in the arable soil amended with carrot residue. Application of organic material is known to reduce the relative abundance of the Acidobacteria. Acidobacteria have been described as slow growing oligotrophs, and well adapted to resource limitation [39] and environments with reduced availability of C substrates [40]. Chloroflexi are also known oligotrophs [41], but little is known about the Gemmatimonadetes. Only one strain of this phylum has been isolated [42], although they must be versatile as they have been detected in different soil ecosystems. Application of carrot residue increased the relative abundance of bacterial groups that are known to be favoured by organic material application, such as the Actinomycetales, Acetobacteraceae (Alphaproteobacteria) and Pseudomonadaceae (Gammaproteobacteria). Strains belonging to the Pseudomonadaceae are known to degrade cellulose [43] and hemicellulose [44], so they were favoured by application of the carrot residue.

Earthworms by actively feeding on the applied carrot residue and mixing it in their gut increased the contact between the soil microorganisms and the organic material- Application of earthworms further increased the relative abundance of Actinomycetales and reduced that of Acidobacteria, Chloroflexi and Gemmatimonadetes compared to the soil amended with carrot residue only. However, they also favoured other groups, such as the Rhizobiales and Rhodobacterales (Alphaproteobacteria), Flavobacteriales (Bacteroidetes) and Lactobacillales (Firmicutes) compared to the soil amended with carrot residue only. Most of these groups were found in the gut of the earthworm *Lumbricus terrestris* L. and are known to be favoured by nutrient rich environments [45].

Application of surfactant decreased the relative abundance of similar bacterial groups as when carrot residue and carrot residue plus earthworms were applied to soil, but the effect was more outspoken. Application of earthworms and surfactant reduced the relative abundance of the same bacterial families (Rubrobacteraceae, Gaiellaceae, Chitinophagaceae and [Kouleothrixeae] and genera (*Rubrobacter* and *Flavisolibacter*). However, application of surfactant favoured other bacterial groups (e.g. Mycobacteriaceae, Sphingobacteriaceae, Bradyrhizobiaceae, Sphingomonadaceae) than when carrot residue was added to soil (e.g. Micrococcaceae, Nocardiaceae, Acetobacteraceae, Pseudomonadaceae). The application of the surfactant increased the microbial activity as evidenced by an increase in CO_2_ emitted from a surfactant-amended soil compared to the unamended soil [9]. Consequently, the surfactant was used as C substrate by the soil microorganisms or the surfactant released physical protected soil organic material that was subsequently metabolized. The organic material added with the surfactant, however, was different from that added with the carrot residue, so it was normal that different bacterial groups were favoured. Although different bacterial groups were favoured by the application of surfactant or carrot residue, it would be difficult to explain that these changes in the bacterial community affected the removal of anthracene from soil. It would mean that the bacteria stimulated by the surfactant lacked the capacity to degrade the anthracene while those stimulated by the carrot residue possessed it.

Soil characteristics might also have been affected by treatment, i.e. application of surfactant, and this in turn might have affected the bacterial community structure. However, application of surfactant had no significant effect on the EC and pH in both the arable and pasture soil (*P* < 0.05). In the unamended arable soil, the pH was 7.5±0.4 at day 56 and 7.0±0.4 in the surfactant-amended soil, while the EC was 1.24±0.06 dS m^-1^ in the unamended soil at day 56 and 1.38±0.02 dS m^-1^ in the surfactant-amended soil. In the unamended pasture soil, the pH was 5.8±0.2 at day 56 and in the surfactant-amended soil 5.4±0.1, while the EC was 1.10±0.01 dS m^-1^ in the unamended soil at day 56 and 1.06±0.02 dS m^-1^ in the surfactant-amended soil. Consequently, these small changes in pH and EC had only a limited effect on the bacterial community structure.

Mixing the soil did not affect the bacterial community structure and the removal of the anthracene was fastest in the mixed soil. Consequently the link between the removal of the contaminant and changes in the bacterial community structure due to treatment, as found in the carrot residue, carrot residue plus earthworms or surfactant amended arable soil, is not straightforward. It appears that an increase in bioavailability of anthracene augmented by mixing had more effect on the removal of the contaminant than biostimulation of the microbial population in the carrot residue amended soil. It has to be remembered that other soil microorganisms, e.g. fungi, have the capacity to remove pollutants from soil and their capacity to mineralize anthracene is well established [46]. Although it is unlikely, they might have been affected in different ways by treatment than the bacteria.

A possible link between changes in the bacterial community structure and removal of anthracene was further weakened when the pasture soil was considered. In the pasture soil, the changes in the taxonomic distributions and in the bacterial community structure were smaller and less significant than in the arable soil, but the removal of anthracene was similar in both soils. Changes in the bacterial community structure in the pasture soil over time were larger than in the arable soil. Why treatment had a smaller, and time a larger effect on the bacterial community in the pasture soil than in the arable soil remains difficult to explain, but some soil characteristics were different between the two soils. The organic C content was twice as high in the pasture than in the arable soil and the silt content was also much higher. The higher organic C in the pasture soil might have ‘buffered’ the bacterial community against changes due to treatment, but not against changes over time.

### Changes in the bacterial groups favoured by the different treatments over time

Increases in the ratio of the relative abundance of bacterial groups when a soil was treated compared to the untreated soil indicates which bacterial groups were favoured by the treatment. For instance, phylotypes belonging to the Nocardiaceae, Streptomycetaceae (mostly *Streptomyces*) and Rhizobiaceae were the first degraders of acetone and/or its metabolites as their relative abundance increased in each of the treatments at day 3, while Streptomycetaceae and Rhizobiaceae in the degradation of anthracene, acetone and/or their metabolites. Phylotypes belonging to these bacterial groups are well known to be involved in the degradation of organic material applied to soil. For instance, Actinobacteria belonging to *Streptomyces* are important degraders of complex carbohydrates in soil [47] and phylotypes belonging to *Bradyrhizobium* and *Rhizobium* (Alphaproteobacteria), and *Arthrobacter* (Micrococcaceae) and *Nocardia* (Nocardiaceae) (Actinobacteria) were identified on decomposing maize leaves [48]. Micrococcaceae participated in the degradation of the carrot and together with Bradyrhizobiaceae in the degradation of the surfactant applied to soil. Phylotypes belonging to the Rhodospirillaceae have been associated with the mineralization of aromatic pollutants (biphenyl, benzoate, and naphthalene) [49] and the anaerobic toluene pathway [50]. In this study, their relative abundance increased in each of the treatments at day 3 so they were first degraders of the organic material applied to soil. Although phylotypes belonging to the Rhodobacteraceae were associated with low-molecular-weight polycyclic aromatic hydrocarbon degradation [51] and their relative abundance increased when anthracene was applied to the arable soil, the sharpest increase was found when carrot or surfactant was applied. Geodermatophilaceae were predominant in desert soil crusts [52]. These actinobacteria are an ecologically significant group, which play a vital role in several biological processes, such as biogeochemical cycles and bioremediation [53]. They were important in the degradation of metabolic products of acetone as their relative abundance increased after 28 days in the acetone-amended soil, but they also participated in the mineralization of the surfactant or its degradation products at day 3. Phylotypes of the Sphingomonadaceae can grow on toluene or *o*-xylene [54] and degrade a wide range of contaminants in soil [55]. In this study, they did not participate in the degradation of acetone or in the initial mineralization of the anthracene, carrot or surfactant, i.e. at day 3, but phylotypes of the Sphingomonadaceae were dominant in the degradation of the metabolic products of the anthracene, carrot but especially the surfactant, at day 14, 28 and 56.

Degradation of acetone, anthracene, carrot and the surfactant applied to pasture soil was done most importantly by the Nocardiaceae and Micrococcaceae. Phylotypes belonging to the Streptomycetaceae also participated in the degradation of the surfactant at day 3 and 14. As such, conditions in the pasture soil limited those bacterial groups that were favoured in the arable soil, e.g. Rhizobiaceae, Rhodobacteraceae and Xanthomonadaceae. It has to be remembered, however, that the microbial community in the pasture soil was larger than in the arable soil as the soil organic matter was nearly twice as high in the pasture than in the arable soil. Microbial biomass C consists generally between 1–3% of the soil organic matter content and is normally higher in a pasture than in an arable soil due to the higher rhizosphere density in the first. It can thus be assumed that the effect of organic material application on the bacterial community structure will be lower in the pasture than in the arable soil.

Degradation of the surfactant and its metabolic products stimulated additional bacterial groups, such as the Bacillaceae, Bradyrhizobiaceae, Geodermatophilaceae and Rhodospirillaceae at day 3, but all of them participated in the degradation.

## Conclusions

The removal of anthracene from soil was not related to changes in the bacterial community structure. Application of earthworms removed most of the contaminant from the arable soil and had a strong effect on the bacterial community structure, while mixing the soil weekly removed all anthracene from the arable soil, but had little or no effect on the bacterial community structure. Application of the non-ionic surfactant inhibited the removal of anthracene from the arable soil compared to the untreated soil and had a strong effect on the bacterial community structure. The removal of anthracene was similar in the arable and pasture soil, but the effect of application of carrot residue, earthworms or surfactant on the bacterial community structure was more outspoken in the arable soil than in the pasture soil. The duration of the incubation altered the bacterial community structure in the pasture soil more than in the arable soil.

## Supporting Information

S1 Fig Schematic overview of the sampling procedure. (TIF)

S2 Fig Heat-map of the most abundant bacterial groups. Abundance of the bacterial groups in the unamended arable (O) and pasture soil (V) (T1), or soil amended with acetone (T2), anthracene (T3), anthracene and mixed every week (T4), anthracene plus carrot residue (*Daucus carota* L.) (T5), anthracene plus carrot residue plus the earthworm *Eisenia fetida* (Savigny, 1826) (T6) or the non-ionic surfactant (Surfynol^®^ 485) (T7) at the onset of the experiment (D0), or incubated aerobically for 3 days (D3), 14 days (D14), 28 days (D28) or 56 days (D56).
(TIF)

S3 Fig The ratio [(Relative abundance of the bacterial group in the treated arable Otumba soil—Relative abundance of the bacterial group in the untreated arable Otumba soil)/Relative abundance of the bacterial group in the untreated arable Otumba soil × 100] of the most important bacterial groups affected positively by the application of maize plants at day 1, 3, 7, 14, 28 and 56 during an aerobic incubation. (TIF)

S4 Fig The ratio [(Relative abundance of the bacterial group in the treated arable Otumba soil—Relative abundance of the bacterial group in the untreated arable Otumba soil)/Relative abundance of the bacterial group in the untreated arable Otumba soil × 100] of the most important bacterial groups affected positively by the application of maize plants at day 1, 3, 7, 14, 28 and 56 during an aerobic incubation. (TIF)

S5 Fig The ratio [(Relative abundance of the bacterial group in the treated arable Otumba soil—Relative abundance of the bacterial group in the untreated arable Otumba soil)/Relative abundance of the bacterial group in the untreated arable Otumba soil × 100] of the most important bacterial groups affected positively by the application of maize plants at day 1, 3, 7, 14, 28 and 56 during an aerobic incubation. (TIF)

S6 Fig The ratio [(Relative abundance of the bacterial group in the treated arable Otumba soil—Relative abundance of the bacterial group in the untreated arable Otumba soil)/Relative abundance of the bacterial group in the untreated arable Otumba soil × 100] of the most important bacterial groups affected positively by the application of maize plants at day 1, 3, 7, 14, 28 and 56 during an aerobic incubation. (TIF)

S7 Fig The ratio [(Relative abundance of the bacterial group in the treated Vera Cruz pasture soil—Relative abundance of the bacterial group in the untreated Vera Cruz pasture soil)/Relative abundance of the bacterial group in the untreated Vera Cruz pasture soil × 100] of the most important bacterial groups affected positively by the application of maize plants at day 1, 3, 7, 14, 28 and 56 during an aerobic incubation. (TIF)

S8 Fig The ratio [(Relative abundance of the bacterial group in the treated Vera Cruz pasture soil—Relative abundance of the bacterial group in the untreated Vera Cruz pasture soil)/Relative abundance of the bacterial group in the untreated Vera Cruz pasture soil × 100] of the most important bacterial groups affected positively by the application of maize plants at day 1, 3, 7, 14, 28 and 56 during an aerobic incubation. (TIF)

S9 Fig The ratio [(Relative abundance of the bacterial group in the treated Vera Cruz pasture soil—Relative abundance of the bacterial group in the untreated Vera Cruz pasture soil)/Relative abundance of the bacterial group in the untreated Vera Cruz pasture soil × 100] of the most important bacterial groups affected positively by the application of maize plants at day 1, 3, 7, 14, 28 and 56 during an aerobic incubation. (TIF)

S10 Fig The ratio [(Relative abundance of the bacterial group in the treated Vera Cruz pasture soil—Relative abundance of the bacterial group in the untreated Vera Cruz pasture soil)/Relative abundance of the bacterial group in the untreated Vera Cruz pasture soil × 100] of the most important bacterial groups affected positively by the application of maize plants at day 1, 3, 7, 14, 28 and 56 during an aerobic incubation. (TIF)
