# Supplementary figures and images for: Changes in the Bacterial Community Structure of Remediated Anthracene-Contaminated Soils
Source: PLoS One. 2016 Oct 11;11(10):e0160991. doi: 10.1371/journal.pone.0160991 (PMC5058544; doi:10.1371/journal.pone.0160991)

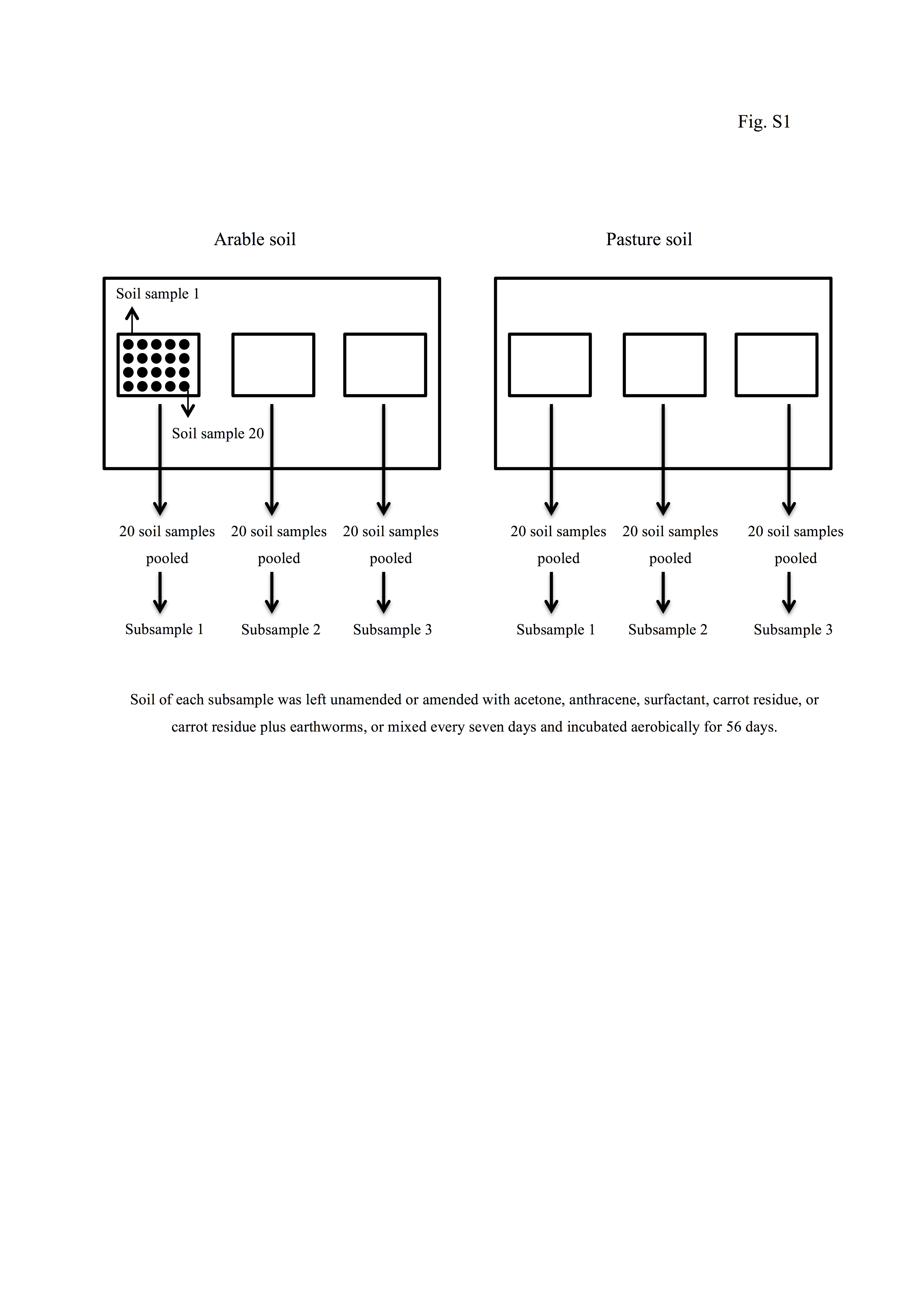

Supplement: S1 Fig — (TIF) [file pone.0160991.s001.tif]

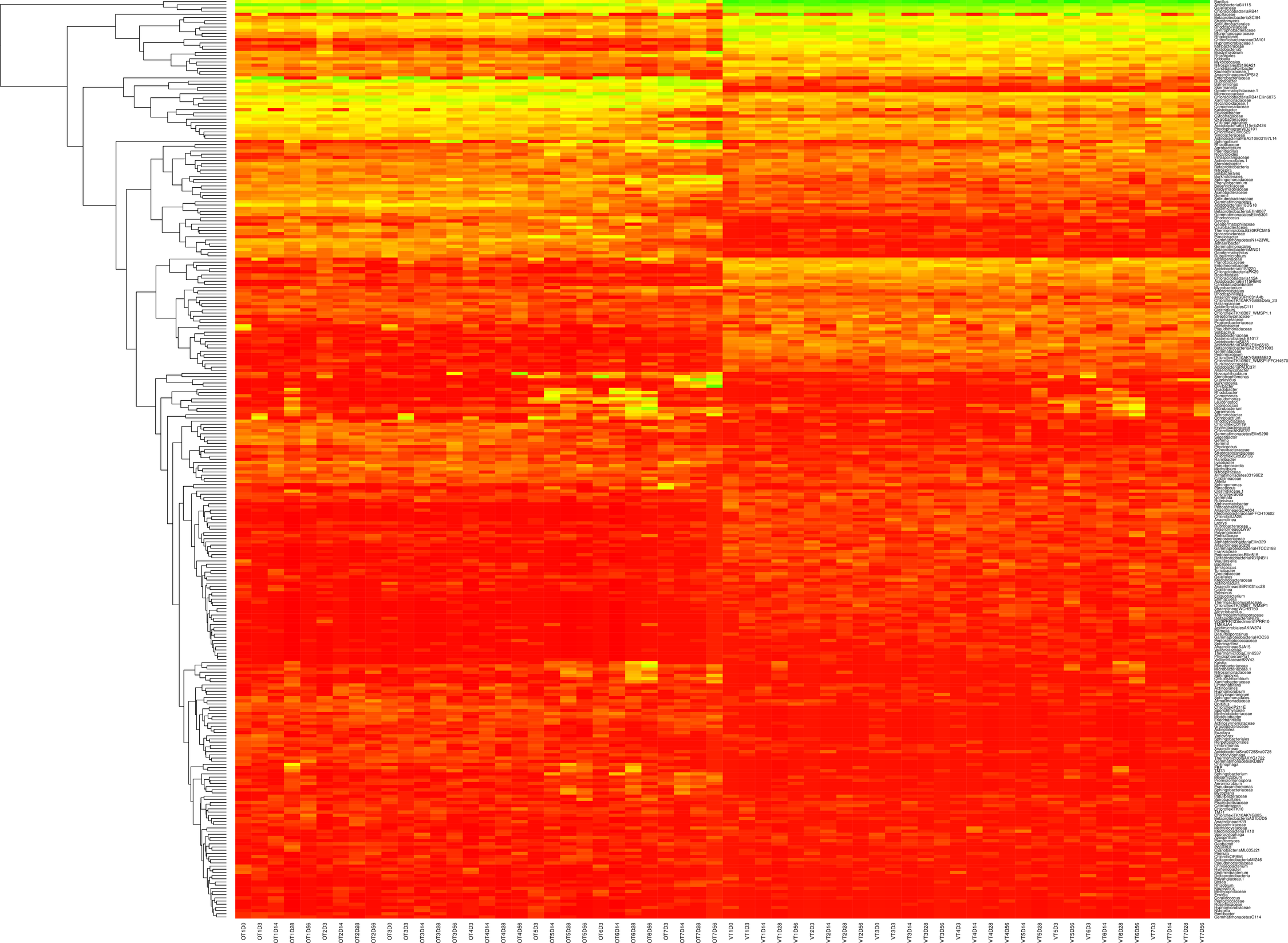

Supplement: S2 Fig — Abundance of the bacterial groups in the unamended arable (O) and pasture soil (V) (T1), or soil amended with acetone (T2), anthracene (T3), anthracene and mixed every week (T4), anthracene plus carrot residue (Daucus carota L.) (T5), anthracene plus carrot residue plus the earthworm Eisenia fetida (Savigny, 1826) (T6) or the non-ionic surfactant (Surfynol® 485) (T7) at the onset of the experiment (D0), or incubated aerobically for 3 days (D3), 14 days (D14), 28 days (D28) or 56 days (D56). (TIF) [file pone.0160991.s002.tif]

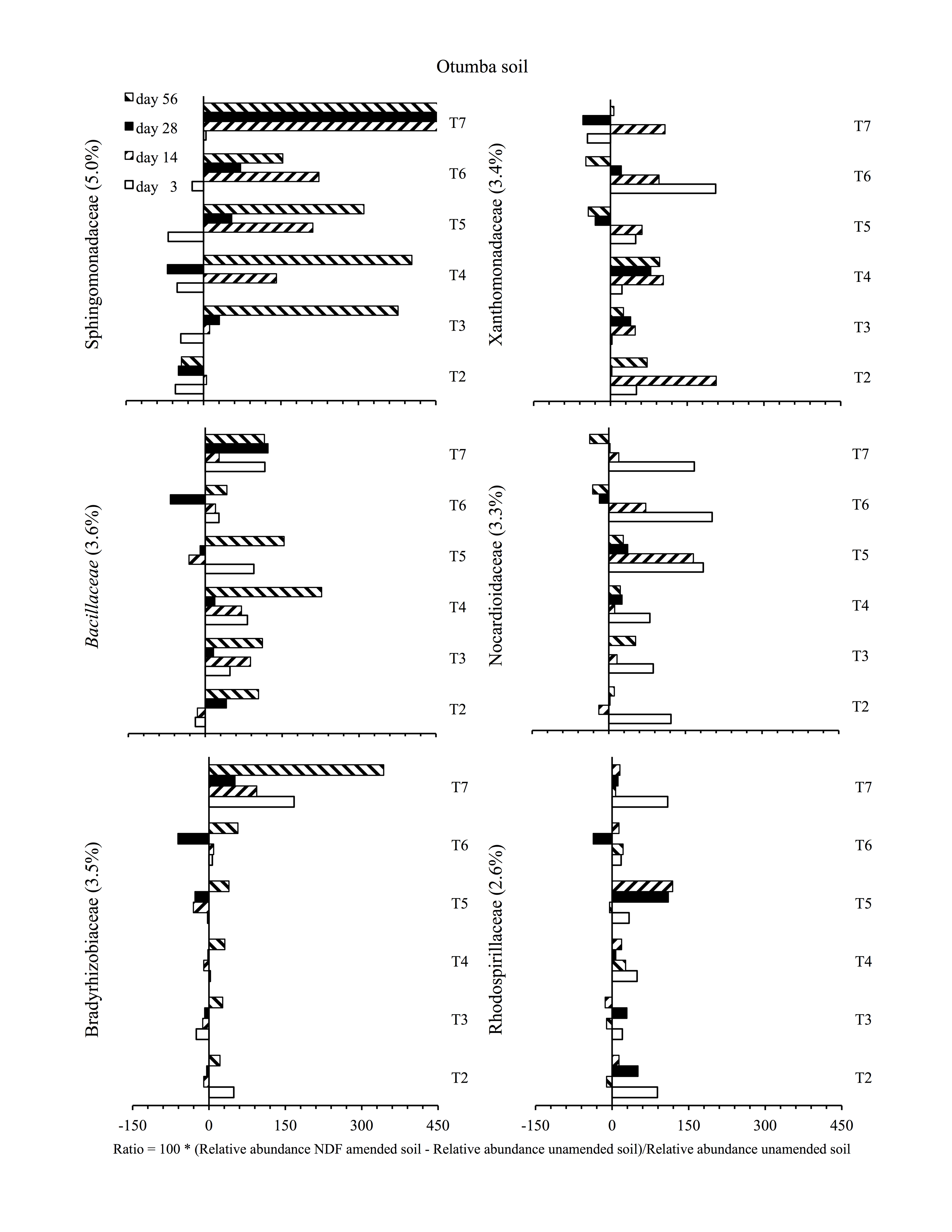

Supplement: S3 Fig — (TIF) [file pone.0160991.s003.tif]

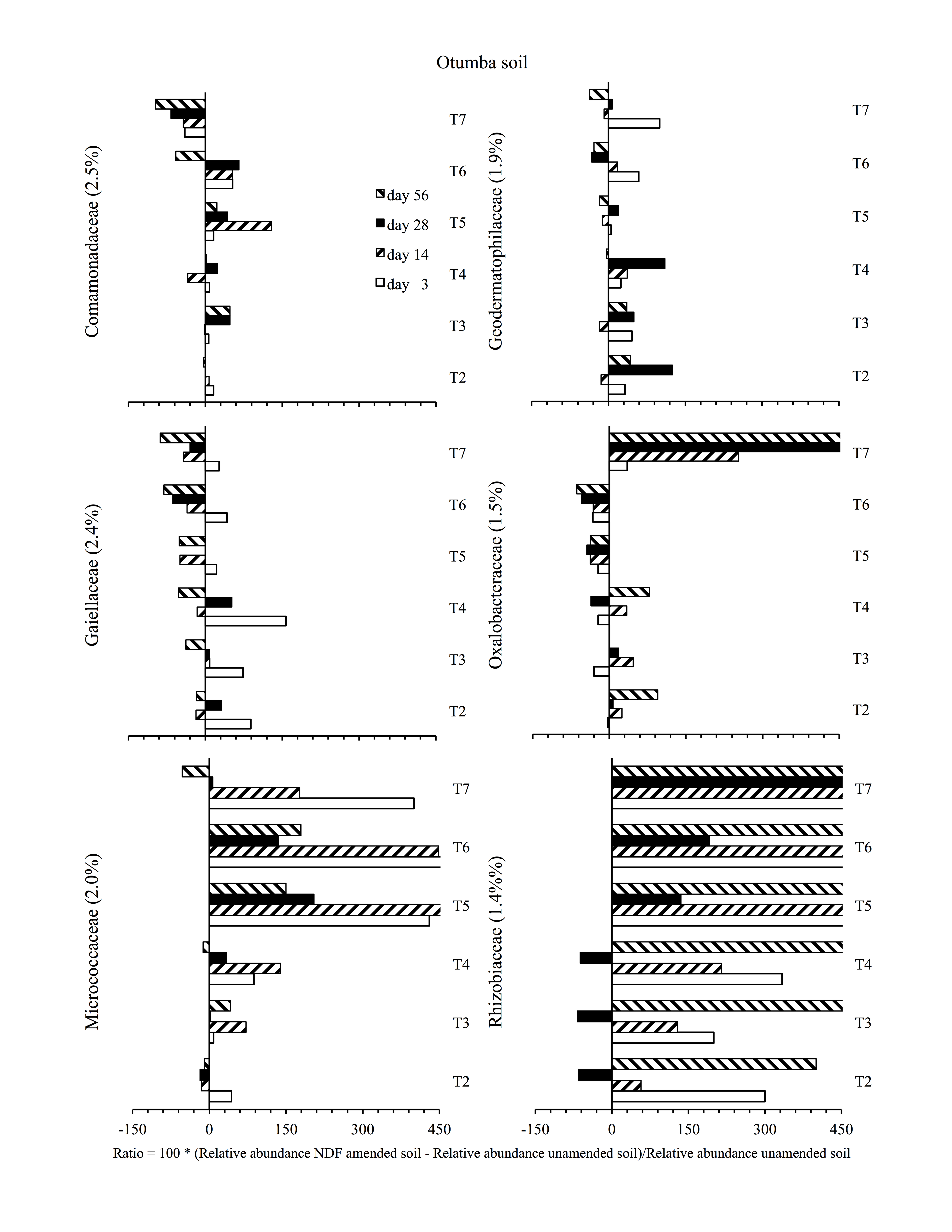

Supplement: S4 Fig — (TIF) [file pone.0160991.s004.tif]

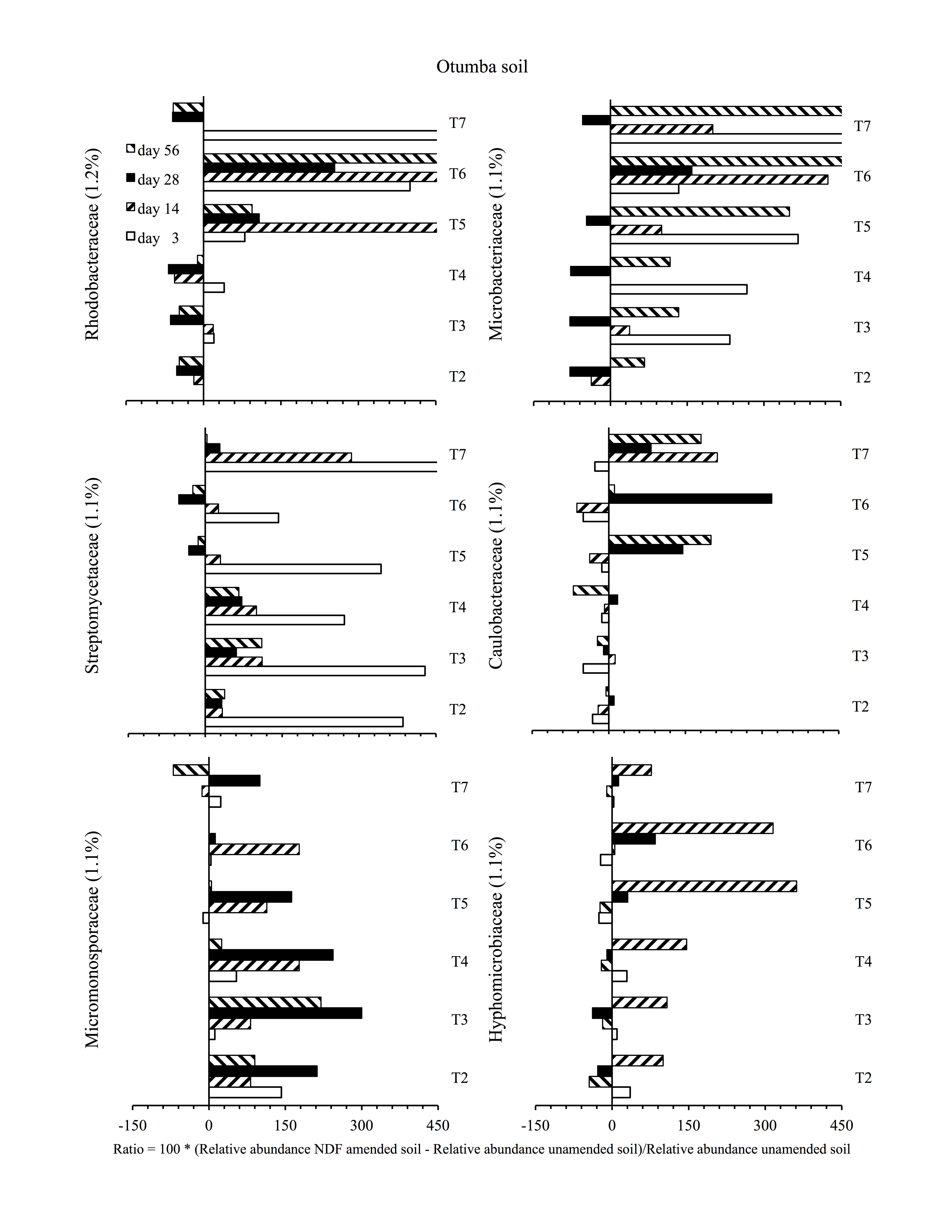

Supplement: S5 Fig — (TIF) [file pone.0160991.s005.tif]

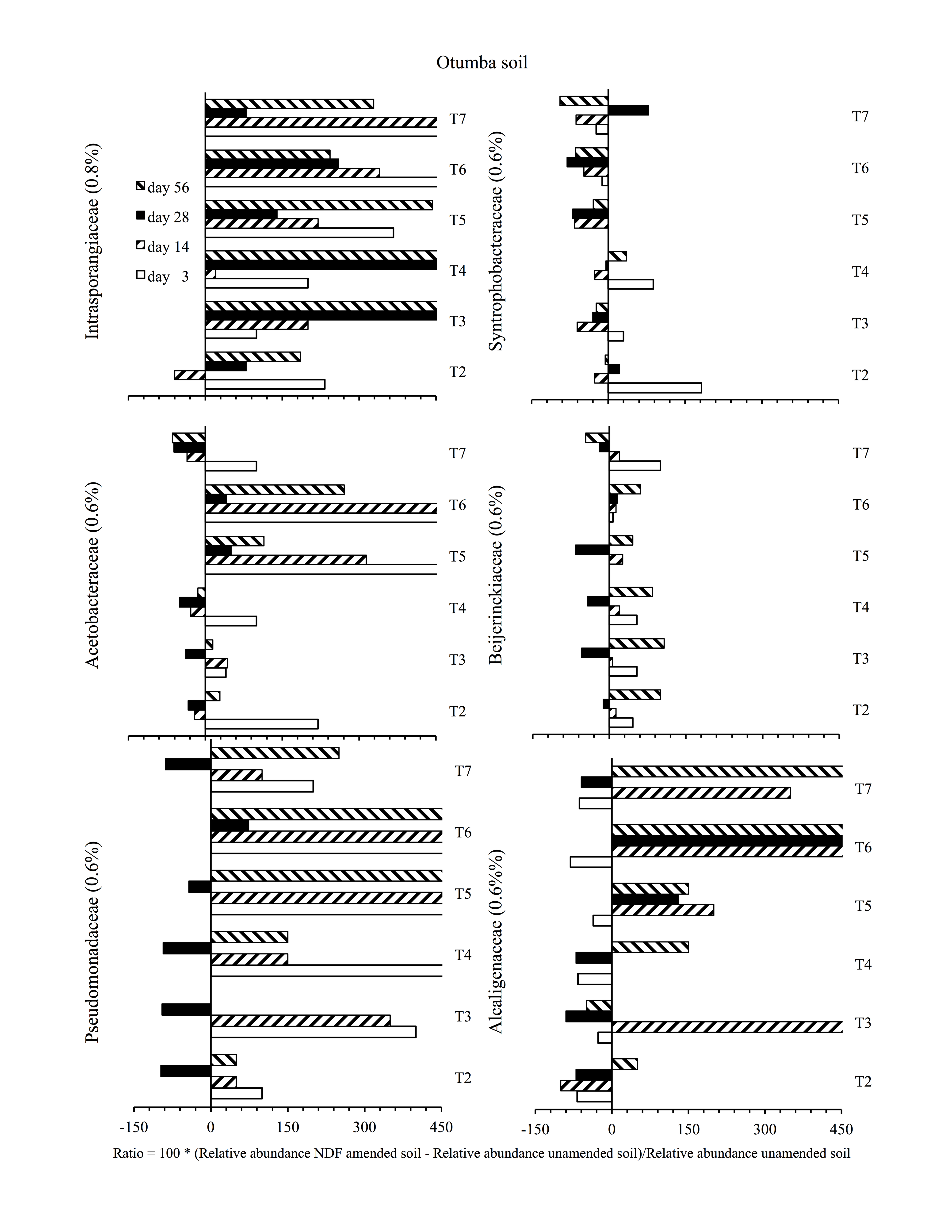

Supplement: S6 Fig — (TIF) [file pone.0160991.s006.tif]

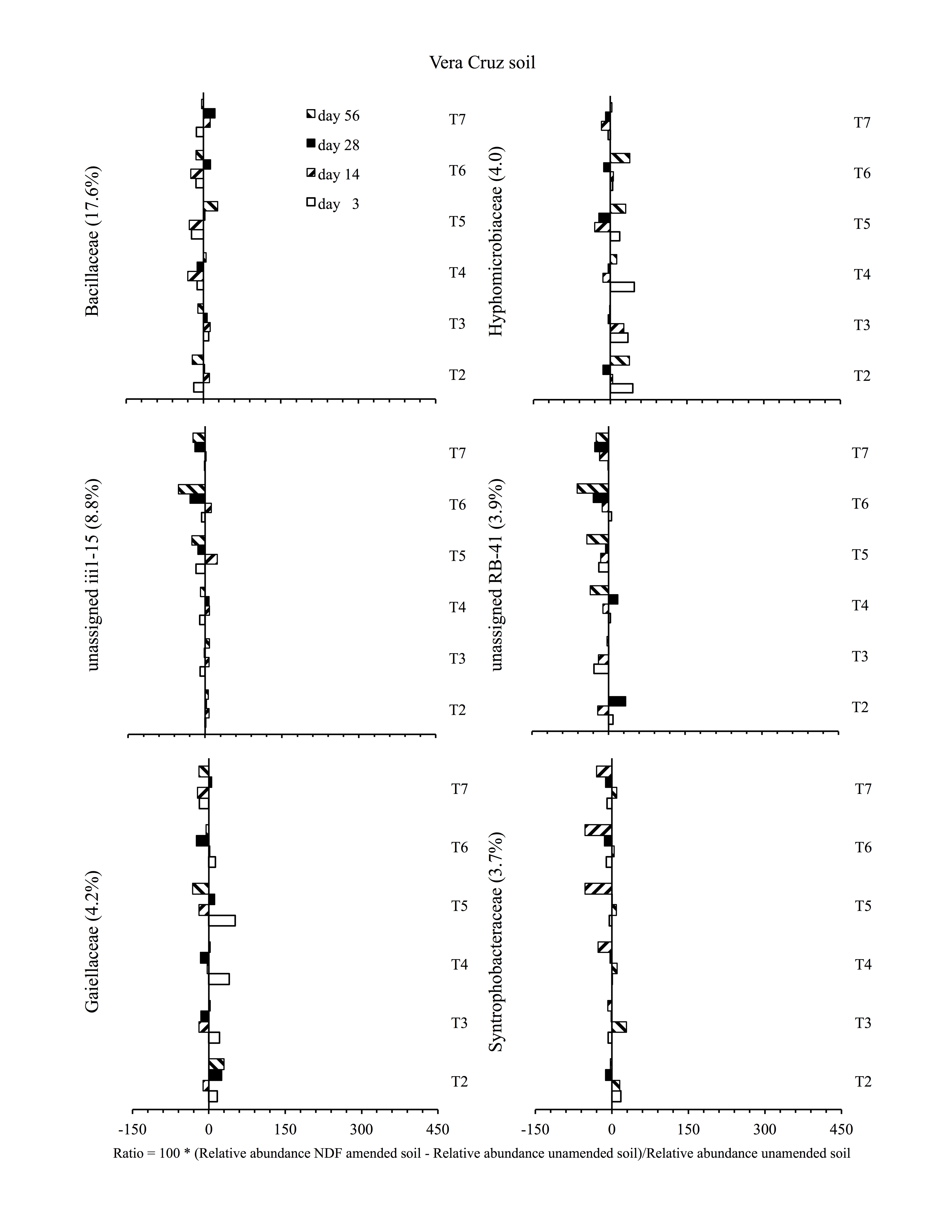

Supplement: S7 Fig — (TIF) [file pone.0160991.s007.tif]

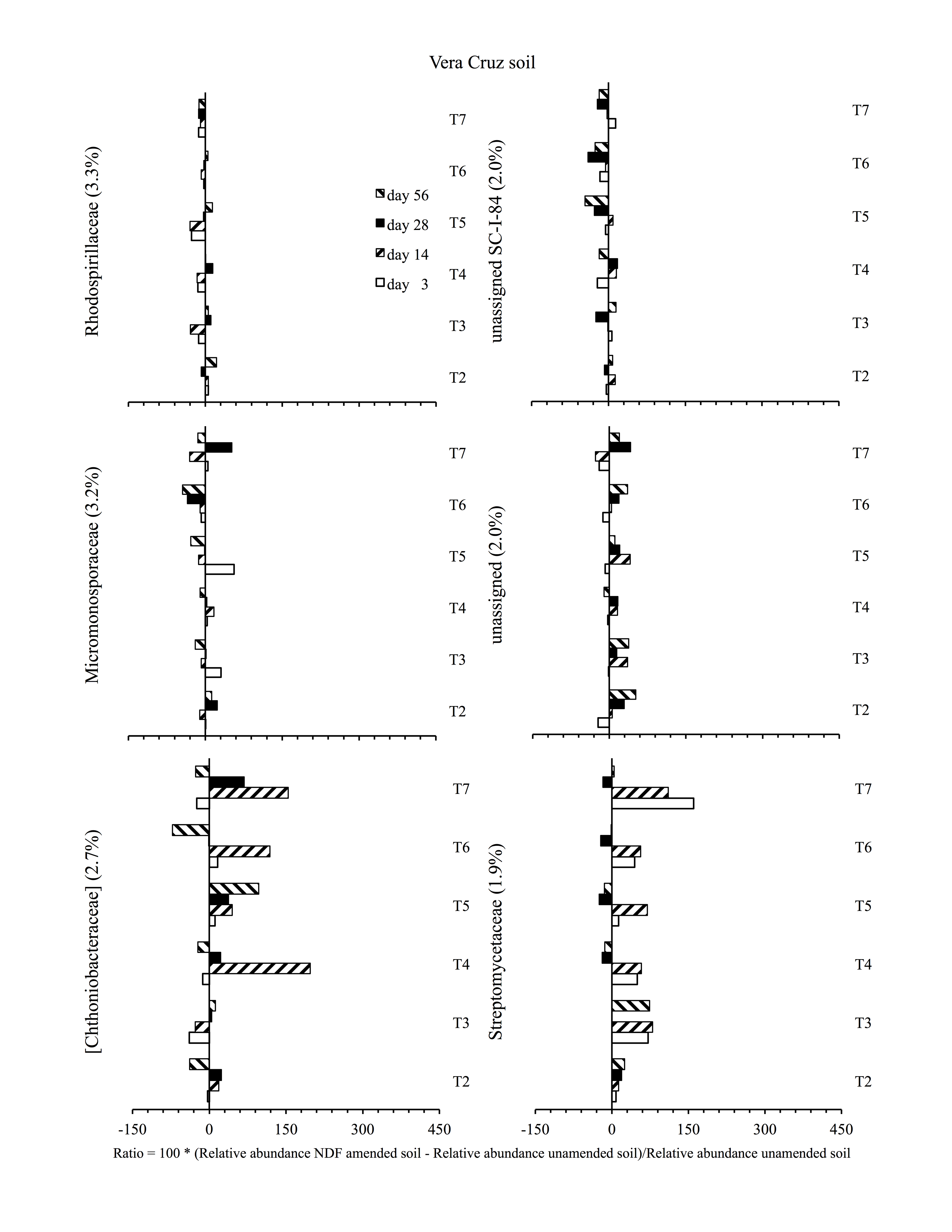

Supplement: S8 Fig — (TIF) [file pone.0160991.s008.tif]

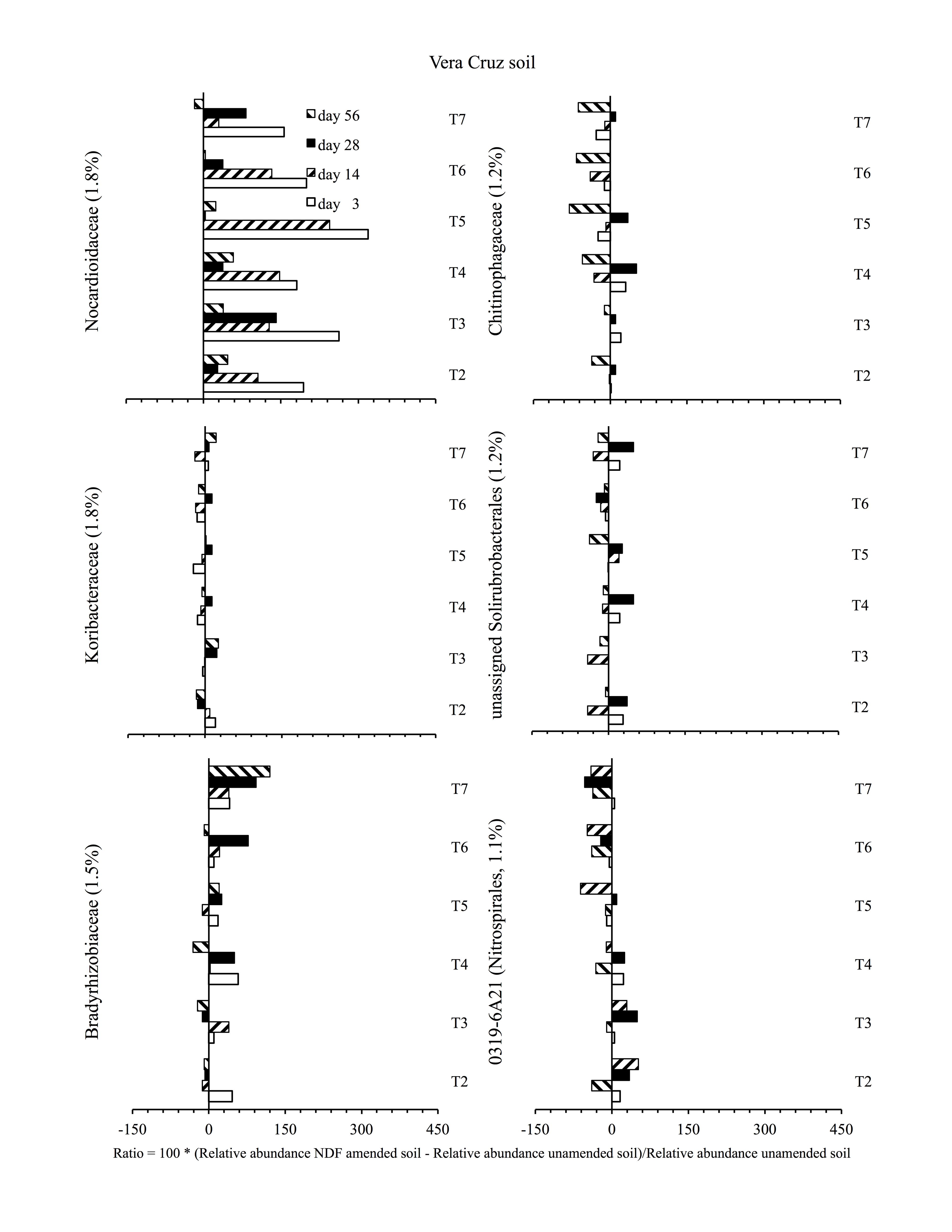

Supplement: S9 Fig — (TIF) [file pone.0160991.s009.tif]

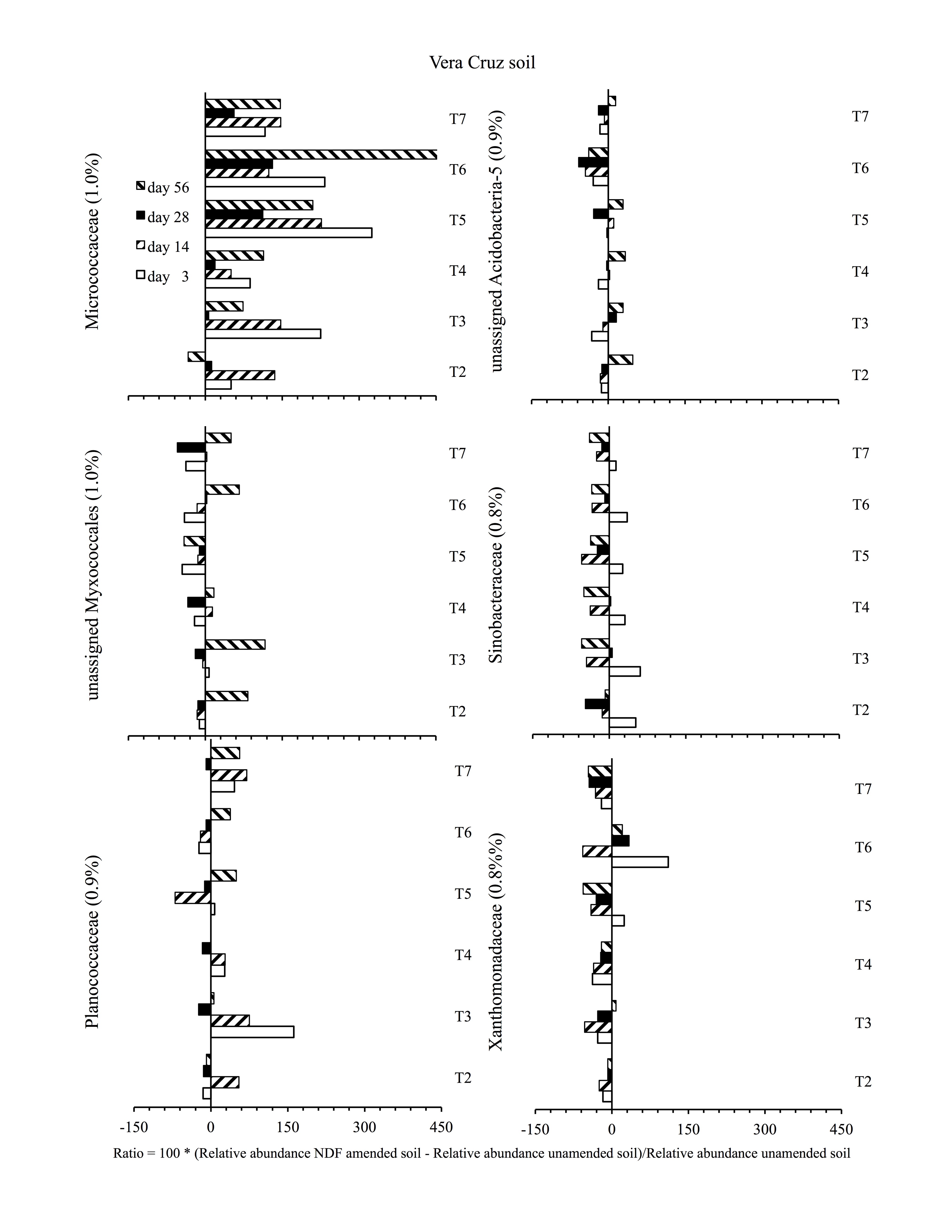

Supplement: S10 Fig — (TIF) [file pone.0160991.s010.tif]
